# Bracken Fern Carcinogen, Ptaquiloside, Forms a Guanine *O*^6^-Adduct in DNA

**DOI:** 10.1021/acs.jafc.4c07187

**Published:** 2025-01-07

**Authors:** Fourat Keskin, Hannah Noone, Mark J. Dickman, Esther Allen, William D. Mulcrone, Lars Holm Rasmussen, Hans Christian Bruun Hansen, Peter J. O’Connor, Andrew C. Povey, Geoffrey P. Margison, David M. Williams

**Affiliations:** † Centre for Chemical Biology, Department of Chemistry, Institute for Nucleic Acids, University of Sheffield, Brook Hill, Sheffield S3 7HF, U.K.; ‡ Department of Chemical and Biological Engineering, University of Sheffield, Mappin Street, Sheffield S1 3JD, U.K.; § Novonesis, Microbe & Culture Research, Bøge Allé 10-12, Hørsholm DK-2970, Denmark; ∥ Department of Plant and Environmental Sciences, University of Copenhagen, Thorvaldsensvej 40, Frederiksberg C DK-1871, Denmark; ⊥ Centre for Occupational and Environmental Health, School of Health Sciences, Faculty of Biology, Medicine and Health, University of Manchester, Manchester M13 9PL, U.K.

**Keywords:** bracken fern, ptaquiloside, carcinogenicity, O6-alkylguanine, MGMT

## Abstract

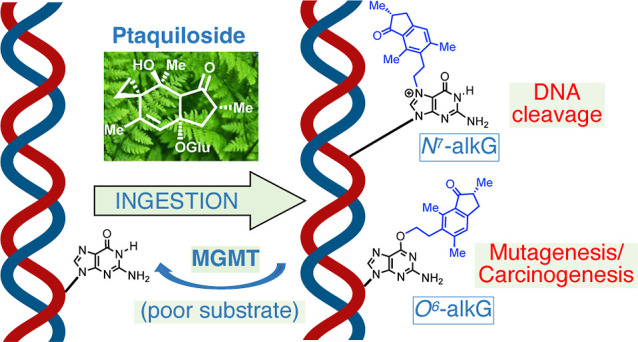

Bracken fern (*Pteridium* sp.) is a viable and vigorous plant with invasive potential, ingestion of which causes chronic illness and cancers in farm animals. Bracken is a suspected human carcinogen, and exposure can result from ingestion of bracken-contaminated water, dairy products, or meat derived from livestock grazing on bracken fern. Bracken is also consumed in the diets of some communities. Ptaquiloside (PTQ), a known bracken carcinogen, is an illudane-type glycoside that forms a highly reactive electrophile, PTQ dienone, known to produce *N*^7^-guanine and *N*^3^-adenine adducts in DNA. Here, we demonstrate for the first time that PTQ dienone also produces an *O*^6^-alkylguanine (*O*^6^-PTBguanine) in DNA. Since *O*^6^-alkylguanines in DNA can be mutagenic, this work provides a potential mechanistic link between PTQ exposure and carcinogenicity. *O*^6^-PTBguanine is poorly repaired by *O*^6^-methylguanine-DNA methyltransferase that acts on other *O*^6^-alkylguanines, further highlighting the potential risk of exposure to bracken and PTQ.

## Introduction

Bracken fern (*Pteridium* sp.) or bracken is an aggressively growing plant found extensively throughout temperate and subtropical areas of the World. The most common species, *Pteridium aquilinum*, is widespread in Europe and covers approximately 1.6% of the UK land mass.^1^ Bracken fern is typically found near woodland, along forest tracks, and on the forest floor below canopy openings. In upland areas, bracken often dominates moors and heathland. Bracken poisoning in farm animals is well-documented^2^ and characterized by a variety of different ailments. These include thiamine deficiency and a depression of bone marrow activity, leading to severe leukopenia and acute hemorrhaging. Chronic bracken poisoning causes progressive retinal degeneration in sheep and is associated with tumors of the upper alimentary tract and bladder in cattle.^2^

**Scheme 1:**
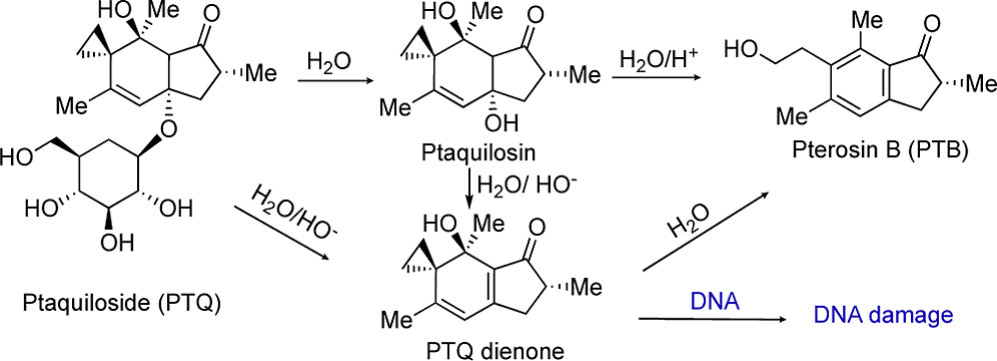
Decomposition of Ptaquiloside to Ptaquilosin, Pterosin B, and PTQ Dienone That Can Cause DNA Damage

The toxicity and carcinogenicity associated with bracken have been known for over 50 years.^3^ The first “active” carcinogen contained in bracken was identified in 1983 as ptaquiloside (PTQ, Scheme 1), an illudane-type glycoside.^4^ PTQ and PTQ-dienone are carcinogenic, but there are no reports of a dose response curve in rats and mice. However, intragastric administration of PTQ to rats has been shown to induce mammary cancer (100%) and ileal tumors (91%) in dose levels of 300 and 380 mg, respectively.^5^ Following the discovery of PTQ, structurally similar illudane glycosides have been found in bracken, of which caudatoside and ptesculentoside are the most well-studied.^6^ Potential routes of human exposure to PTQ and other illudane glycosides include contaminated surface water, soil, and groundwater in bracken-infested areas.^2^ Field studies demonstrate substantial PTQ wash-off from bracken during rain events, resulting in elevated PTQ concentrations in pore water, eventually resulting in contamination of surface and groundwater, including drinking water reservoirs. Exposure through dietary routes include the milk of grazing cattle and the meat of calves fed on a diet containing bracken.^7^ In several parts of the world, bracken is consumed by humans, particularly bracken crosiers harvested in spring.^8^ Exposure may also take place via inhalation of spores^9^ and by skin exposure to bracken leaves, but the risk is hardly quantified. However, although bracken is designated by the WHO/IARC as a possibly carcinogenic to humans,^10^ detailed studies supporting this or indeed the consequences of human exposure to PTQ are not available.

PTQ seems to be the most abundant illudane glycoside^11^ and is highly soluble in water and relatively stable at neutral or slightly acidic pH. However, below pH 4, PTQ has a half-life of just a few hours, undergoing elimination of glucose and formation of ptaquilosin (Scheme 1). Hydrolysis of ptaquilosin produces pterosin B (PTB).^12−14^ At alkaline pH, PTQ decomposition occurs in minutes, forming PTQ dienone^12−14^ (Scheme 1). PTQ dienone contains a highly electrophilic spirocyclopropane moiety adjacent to a hydroxyl group. It reacts rapidly with a variety of nucleophiles including DNA bases, undergoing aromatization in the process^12,15^ The reported mutagenicity of PTQ at slightly alkaline pH has been attributed to its conversion to PTQ dienone,^16^ whose carcinogenicity has also been established.^17^

**Figure 1:**
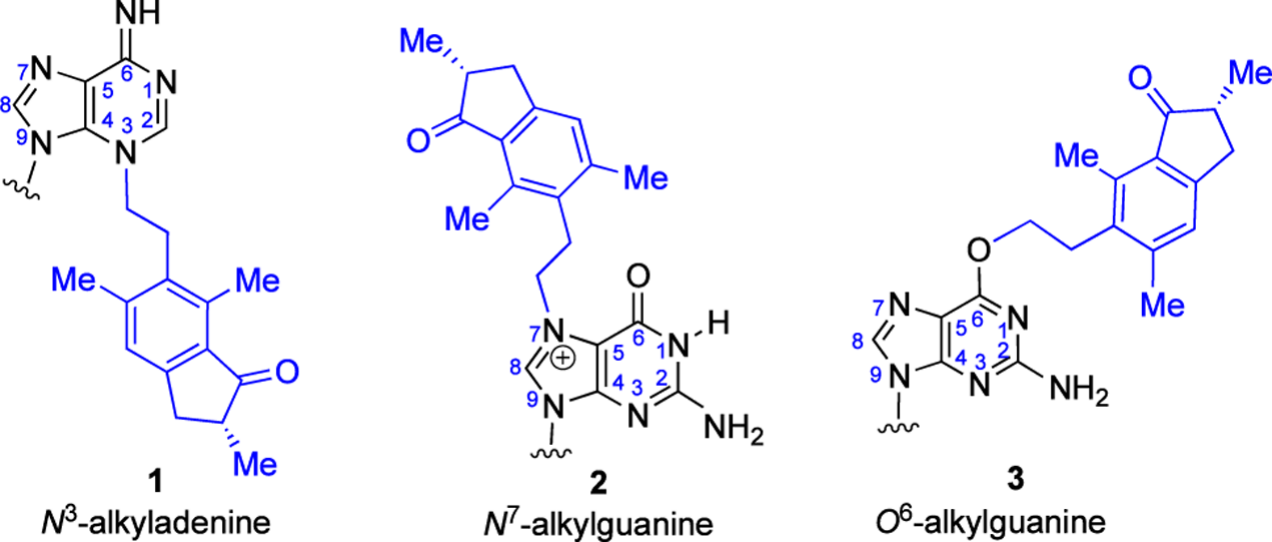
Structures of alkylated adenine and guanine formed following the reaction of DNA or guanosine with PTQ dienone: 1 and 2 have been identified in reactions with DNA^20^ following thermal hydrolysis/depurination and 2 and 3 have been identified in the reaction of PTQ dienone with guanosine.^12^

The reactivity of PTQ dienone with selected amino acids, ribonucleosides, and the ribonucleotides AMP and GMP has been described by Yamada and co-workers.^12^ Alkylation of the heterocyclic bases of the ribonucleosides resulted in products derived from the reaction of uridine (N3 and O4 atoms), cytidine (N3 atom), guanosine (N7 and O6), and adenosine (N6). However, in the latter instance, this most probably results from N1-alkylation of the adenine ring followed by a Dimroth rearrangement.^18^ Interestingly, no products derived from the alkylation of adenosine on the N3 position were observed. In DNA, many of these nucleophilic sites are less accessible due to the double-helical structure. Thus, in DNA duplexes, alkylation of cytosine or the N6 amino group of adenine is generally not observed.^19^ Subsequent studies from the Yamada group demonstrated the cleavage of double-stranded plasmid DNA following treatment with PTQ dienone (but not PTQ) and subsequent heating. Hydrolysis of the DNA was attributed to alkylation at adenine and guanine sites, followed by depurination and chain cleavage at the resulting abasic sites.^15,20^ Following treatment of salmon sperm DNA^15^ with PTQ dienone and subsequent heating, the alkylated *N*^3^-alkyladenine (*N*^3^-alkA) and *N*^7^-alkylguanine (*N*^7^-alkG) adducts 1 and 2 (Figure 1), respectively, were identified, with the latter being formed in a 3-fold excess. When the short oligodeoxyribonucleotide d(ACGT)^20^ was treated with PTQ dienone, the major product obtained was PTB, together with unreacted d(ACGT) and a number of other DNA-derived products. The two most abundant products were isolated by HPLC and further characterized. Thus, heating these samples resulted in depurination of the alkylated purine-containing nucleosides, releasing *N*^7^-alkG and *N*^3^-alkA in a ratio of 2.4:1. Although *N*^7^-alkG is the major product in this reaction, it should also be noted that hydrolysis of *N*^7^-alkGs in DNA can lead to the formation of imidazole ring-opened products through attack of water at C8.^21^ These are much more stable toward hydrolysis at the glycosylic linkage than *N*^7^-alkyl-2′-deoxyguanosines, so this may have led to an underestimation of the initial amount of this adduct. Furthermore, these studies would not identify other expected adducts such as *O*^6^-alkylguanines since these are not labile under the experimental conditions used for glycosylic bond hydrolysis.

*N*^7^-AlkGs in DNA are neither toxic nor mutagenic per se, while *N*^3^-alkAs can be toxic via blocking DNA replication.^22^ The half-lives for depurination of *N*^3^-alkA and *N*^7^-alkG (methyl and ethyl adducts) from DNA are approximately 6.5 and 155 h, respectively,^23^ and the abasic sites generated can be mutagenic.^24^ In contrast, *O*^6^-alkGs in DNA are significantly more stable: comparative studies of nucleoside stabilities show no decomposition of *O*^6^-MedG over 11 days, conditions under which over 80% depurination of *N*^7^-MedG occurs.^25^ Furthermore, *O*^6^-alkGs can be both toxic and mutagenic, typically miscoding with thymine during DNA replication, producing GC → AT transition mutations.^26^ Recently, two mutational signatures have been described in urothelial tumors from cattle that grazed on pastures with bracken fern and human cell lines treated with bracken fern extracts and ptaquiloside.^27^ One signature contains mutations at GC base pairs and is thus potentially consistent with the formation of an *O*^6^-alkG adduct, while the second is defined by a preponderance of mutations at AT base pairs. The latter mutations may arise from already described PTQ-adenine adducts^20^ but are also consistent with the formation of *O*^4^-alkT adducts, which are likely to be formed as well.

The relative amounts of *O*^6^-alkGs formed in DNA depend on the nature of the alkylating agent.^28^ In this context, highly reactive or hard electrophiles^29^ show increased levels of alkylation at guanine O6 compared to less reactive or soft electrophiles that preferentially react at the soft electrophilic N7 position. *N*-alkyl-nitrosoureas, that alkylate via hard electrophilic diazonium species, result in a typical ratio for ethylation on guanine O6 versus N7 of 0.65 and around 1.7 for larger secondary alkyls such as isopropyl.^30^ Previously, the formation of an *O*^6^-alkG adduct following the reaction of PTQ dienone has been demonstrated for the ribonucleoside guanosine^12^ but not for DNA. However, the formation of an *O*^6^-alkG in DNA following exposure to PTQ dienone would be consistent with the observed mutagenicity and carcinogenicity associated with exposure to PTQ dienone and PTQ. Furthermore, the genotoxicity of this putative adduct is likely to depend on its repair by *O*^6^-methylguanine-DNA methyltransferase (MGMT). MGMT repairs many types of *O*^6^-alkGs in DNA via irreversible transfer of the alkyl group to Cys145.^26^ However, we^31^ and others^32^ have shown that certain *O*^6^-alkG adducts are very poor substrates for MGMT. If such damage is not processed by other repair pathways, it is likely to persist in DNA and thereby be more harmful.

The objective of this study was to provide evidence for the first time that exposure to PTQ derived from bracken fern can form *O*^6^-alkG adducts in DNA. Since such adducts are likely to be carcinogenic, such characterization would provide a basis for the observed carcinogenicity resulting from PTQ exposure. We have also explored the potential harmful effects of adduct formation in humans by investigating the ability of the DNA damage reversal protein MGMT to repair such adducts.

## Materials and Methods

### Chemicals, Reagents, and Equipment

All of the reagents were obtained from commercial suppliers and used without further purification. Ptaquiloside was isolated as described.^11^ Dry solvents were obtained from the University of Sheffield Grubbs apparatus, and all anhydrous reactions were carried out in a flame-dried apparatus under N_2_ using standard Schlenk techniques unless otherwise stated. Column chromatography was performed on silica gel for flash chromatography (30–70 μm). Thin layer chromatography (TLC) was performed on precoated Merck silica gel 60 F254 aluminum backed plates. TLC chromatograms were visualized under UV (254 nm). NMR spectra were recorded on a Bruker AVANCE 3 HD 400 spectrometer, and chemical shifts are reported in δ values relative to tetramethylsilane as an external standard. J values are given in Hz. Mass spectrometry was either performed by the University of Sheffield Mass Spectrometry Service using the method of positive electrospray ionization on a Agilent 1260 Infinity liquid chromatography instrument connected to an Agilent 6530 Q-ToF, using an Agilent Zorbax C18 2.1 mm × 50 mm, 1.8 μm column at a flow rate of 0.4 mL/min, or by the University of Sheffield Chemical and Biological Engineering department Orbitrap Exploris 240 LC–MS system using a Phenomenex Gemini C18 5 μm 4.6 × 250 mm column, with a flow rate of 0.75 mL/min. Analytical reverse phase high-performance liquid chromatography (RP-HPLC) was performed on a Transgenomic Wave 3500 HPLC system using a Phenomenex Gemini C18 5 μm 4.6 × 250 mm column, at 40 °C and a flow rate of 1 mL/min. UV detection was recorded at 260 nm unless specified otherwise. DNA synthesis was carried out using an Applied Biosystems Incorporated 394 DNA synthesizer using standard DNA.

### Chemical Synthesis

The following were synthesized as described: pterosin B,^33^*N*^7^-methyl-2′-deoxyguanosine,^34^ and *O*^6^-methyl-2′-deoxyguanosine.^35^

### *O*^6^-(Pterosin B)-2′-deoxyguanosine (*O*^6^-PTB-2′-deoxyguanosine) (5)

*O*^6^-Mesitylenesulfonyl-3′,5′-*bis*-*O*-(*t*-butyldimethylsilyl)-2′-deoxyguanosine (4)^35^ (12.4 mg, 18 μmols), pterosin B (10 mg, 46 μmols, 2.5 equiv), and DABCO (8.2 mg, 73.4 μmols, 4 equiv) were placed under Ar in a flask sealed with a rubber septum. Dry 1,2-DME (300 μL) was then added, and the mixture was stirred for 1 h at room temperature (rt). DBU (11 μL, 73 μmol) was then added, and the mixture was stirred for 2 days, turning the white precipitate to a yellow solution. The solvent was then evaporated to give the *O*^6^-(pterosin B)-3′,5′-*bis*-*O*-(*t*-butyldimethylsilyl)-2′-deoxyguanosine intermediate as a yellow oil. *R*_f_ (EtOAc) = 0.75, PTB: *R*_f_ (EtOAc) = 0.51.

The intermediate was redissolved in THF (300 μL) and sealed with a rubber septum, TBAF in THF (1 M, 11.6. μL, 40.3 μmol) was added, and the mixture was stirred with an O/N mixture at rt. The solvent was evaporated, and the crude product was purified by RP-HPLC (5–65% MeCN/water over 30 min) to afford pterosin B and the crude product as a white solid. The crude product was further purified by preparative TLC (10% MeOH/EtOAc) to afford the product (5) as a white solid (1.5 mg, 18%). ^1^H NMR (400 MHz, DMSO-*d*_6_): δ 8.10 (s, 1H), 7.22 (s, 1H), 6.37 (s, 2H), 6.22 (dd, *J* = 7.8, 6.0 Hz, 1H), 5.27 (d, *J* = 4.0 Hz, 1H), 4.99 (t, *J* = 5.6 Hz, 1H), 4.51 (t, *J* = 7.4 Hz, 2H), 4.36 (dddd, *J* = 6.2, 4.0, 3.0, 2.6 Hz, 1H), 3.83 (ddd, *J* = 5.0, 4.7, 2.6 Hz, 1H), 3.58 (ddd, *J* = 11.6, 5.6, 5.0 Hz, 1H), 3.51 (ddd, *J* = 11.6, 5.6, 4.7 Hz, 1H), 3.24 (dd, *J* = 16.7, 7.7 Hz, 1H), 3.17 (t, *J* = 7.4 Hz, 2H), 2.68 (s, 3H), 2.66–2.54 (m, 3H), 2.49 (s, 3H), 2.22 (ddd, *J* = 13.0, 6.0, 3.0 Hz, 1H), 1.17 (d, *J* = 7.3 Hz, 3H); ^13^C NMR (126 MHz, DMSO-*d*_6_): δ 209.73, 160.59, 160.15, 154.39, 153.04, 144.91, 138.34, 137.54, 134.92, 131.90, 126.22, 114.40, 88.08, 83.25, 71.23, 64.54, 62.21, 55.39, 42.40, 33.66, 28.19, 21.35, 16.67, 13.71; *R*_f_ (10% MeOH/EtOAc) = 0.38, PTB: *R*_f_ (10% MeOH/EtOAc) = 0.82; HRMS *m*/*z*: (ESI^+^) [M + H]^+^ calcd for C_24_H_49_N_5_O_5_, 468.22541; found, 468.22344; HPLC retention time: 29.51 min, 3–75% MeCN in H_2_O over 36 min.

### Oligodeoxyribonucleotide (ODN) Methods

**Figure 2:**
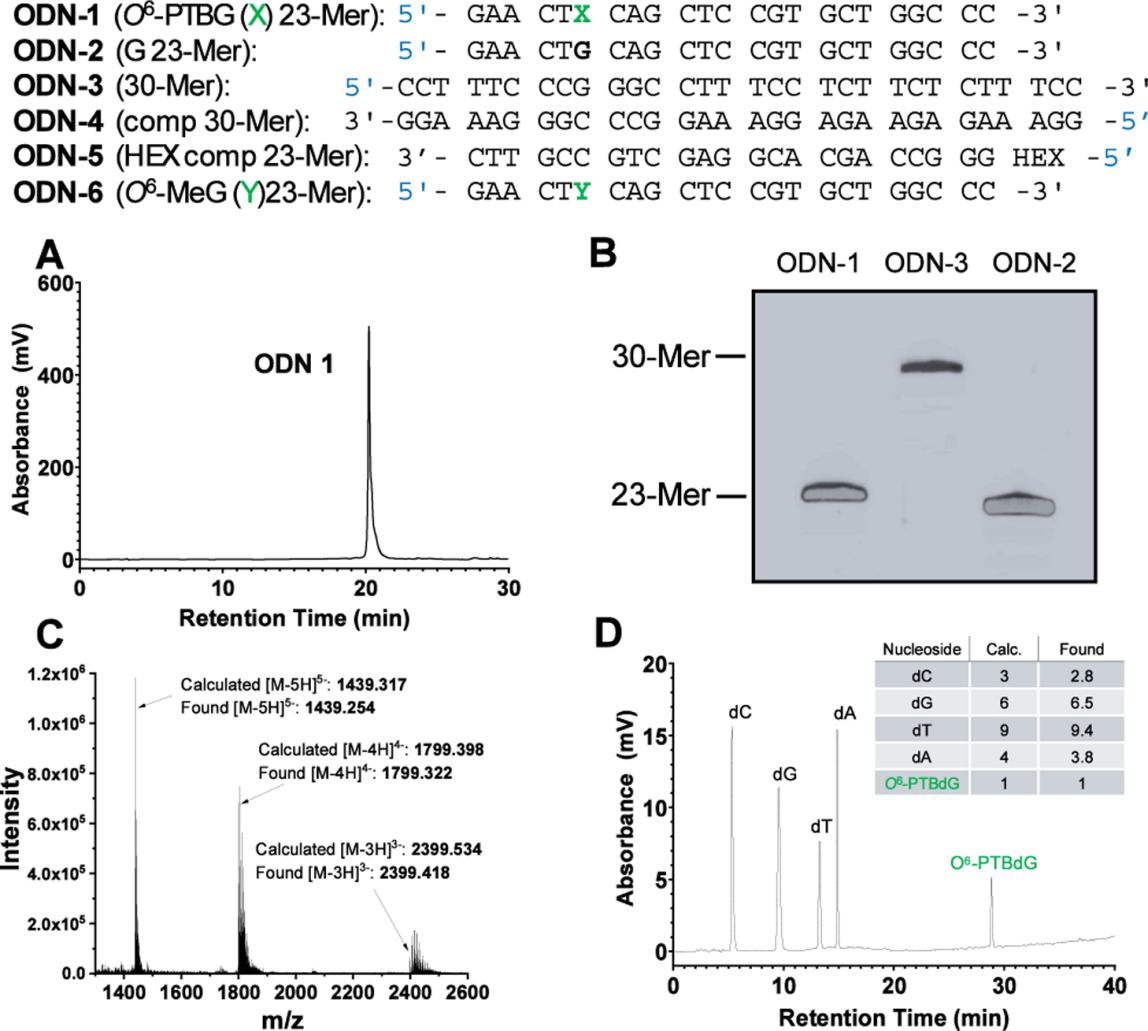
ODNs used and characterization of *O*^6^-PTBG-containing ODN: (A) RP-HPLC chromatogram of purified ODN-1, 12.5–25% MeCN in 0.1 M TEAA pH 7.0 over 30 min; (B) 20% denaturing PAGE stained with Sybr Gold; (C) ESI MS of ODN-1; theoretical mass = 7201.627 Da, experimental mass = 7201.302 Da; and (D) nucleoside composition analysis of ODN-1 5′-GAACT(*O*^6^-PTBG]CAGCTCCGTGCTGGCCC) (see Table S1 in the Supporting Information).

All of the ODNs (Figure 2) except for ODN-1 (containing *O*^6^-PTBG) were purchased from ATD-Bio purified by HPLC.

### Synthesis of ODN-1

ODN-1 was prepared from the corresponding 2-amino-6-methylsulfonylpurine (X)-containing sequence 23 mer ODN sequence (5′-GAA CTX CAG CTC CGT GCT GGC CC) that was synthesized as described.^31^ One controlled pore glass (CPG) column (1 μmol) of this 2-amino-6-methylsulfonylpurine-containing ODN was dried in a desiccator before treatment with 10% DBU in dry MeCN (5 mL) for 3 min, and the column was then washed with MeCN (15 mL). The column was then dried by blowing argon through, and the CPG was then transferred into a Wheaton Screw-Top V-Vial (Sigma-Aldrich) (2 mL). A solution of pterosin B (10 mg) in 10% DBU/dry MeCN (500 μL) was then added to the vial which was flushed with argon, and the mixture was shaken for 5 days at rt. Concentrated aqueous ammonia solution (33%, 1 mL) was then added, and the mixture was shaken for a further 3 days at rt. The supernatant was removed from the CPG beads, which were then washed with water (3 × 300 μL). Unreacted pterosin B was then extracted using ethyl acetate (3 × 3 mL). ODN-1 was purified by RP-HPLC using a Phenomenex Gemini C18 5 μm 4.6 mm × 250 mm column at a flow rate of 1 mL/min. Gradient 12.5–25% MeCN in 0.1 M TEAA, pH7.0 over 30 min. Retention time 20.0 min. ESI-MS Calcd 7201.627, found 7201.302.

### Nucleoside Composition Analysis of ODN-1

ODN-1 was digested using the Nucleoside Digestion Mix (10 μL) from New England BioLabs in Milli-Q water (160 μL) and 10X Nucleoside digestion mix buffer (20 μL) and then incubated at 37 °C for 1 h. The mixture was then analyzed using RP-HPLC using a Phenomenex Gemini C18 5 μm 4.6 mm × 250 mm column, at 40 °C and a flow rate of 1 mL/min. The gradient used was 3–75% MeCN/H_2_O over 36 min (Figure 2D) in the main text. Nucleoside composition of ODN-1 was calculated from the peak areas of the respective nucleosides in the HPLC trace (Table S1 in the Supporting Information).

### Preparation of PTQ Dienone

To a sample of PTQ (4 mg) was added 0.02 M aqueous Na_2_CO_3_ (2 mL), which was left stirring for 20 min at rt. PTQ dienone was extracted using a solution of 2:1 hexane/diethyl ether (3 × 3 mL). Formation of PTQ dienone was confirmed by HPLC and MS (see Figure S1 in the Supporting Information). The organic layer was passed through a plug of Na_2_CO_3_ before rotary evaporation.

### Reaction of PTQ Dienone with Duplex DNA

**Figure 3:**
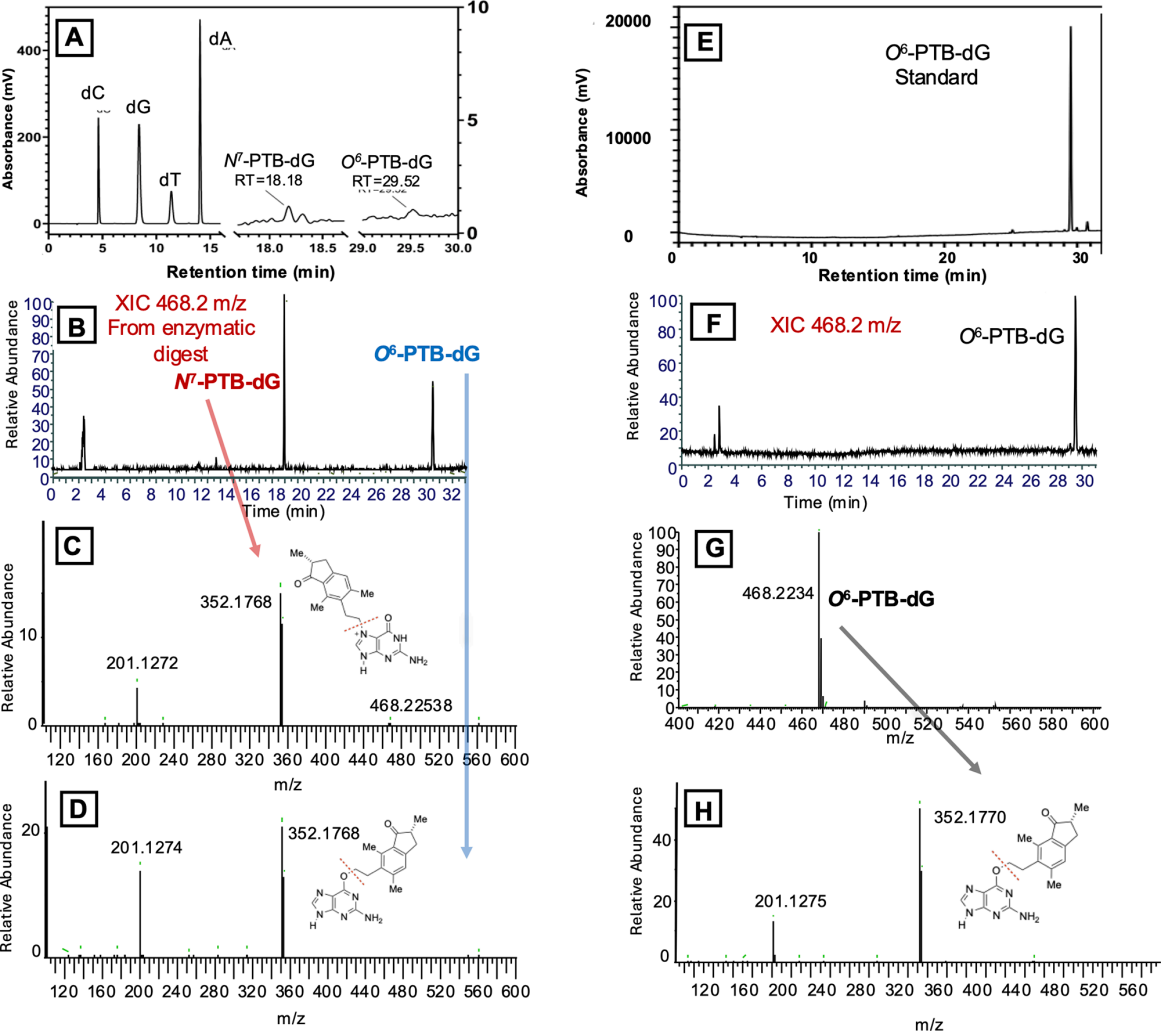
Nucleoside composition analysis of the PTQ dienone-treated DNA duplex: (A) RP-HPLC following enzymatic digestion of PTQ dienone-treated DNA (see the Supporting Information for details); (B) extracted ion chromatogram (XIC) of the PTQ dienone-treated DNA duplex following enzymatic digestion to nucleosides and XIC of *N*^7^-PTB-dG/*O*^6^-PTB-dG 468.2 [M + H]^+^ (*m*/*z*); (C) MS/MS spectrum of selected ion 468.2 [M + H]^+^ (*m*/*z*), LC retention time 18.18 min; (D) MS/MS spectrum of selected ion 468.2 [M + H]^+^ (*m*/*z*), LC retention time 29.52 min; (E) RP-HPLC analysis of the *O*^6^-PTB-dG standard; (F) XIC of the 468.2 [M + H]^+^ (*m*/*z*) *O*^6^-PTB-dG standard shown in panel E; (G) MS of the *O*^6^-PTB-dG standard; and (H) MS/MS spectrum *O*^6^-PTB-dG. This corresponds to that shown in panel D, confirming the identity of *O*^6^-PTB-dG from the nucleoside digest. In panels C, D, and H, the masses of 352.17 and 201.127 *m*/*z* values are obtained from expected fragmentation across the glycosidic bond and pterosin B moiety, respectively, as shown.

PTQ dienone (obtained as described above) was redissolved in MeCN (250 μL) and added to 100 nmol of an annealed duplex of ODN-3 and ODN-4 in 0.1 M phosphate buffer (750 μL, pH 7.5). The mixture was then incubated at 37 °C for 2 h. Unreacted pterosin B was removed by extraction into EtOAc (3 × 500 μL), before desalting using a Cytiva NAP-10 gel filtration column. Ten micrograms (0.53 nmol, 10 μL) of the dienone-exposed duplex DNA was then digested using the Nucleoside Digestion Mix (10 μL) from New England BioLabs in Milli-Q water (160 μL) and 10X Nucleoside digestion mix buffer and then incubated at 37 °C for 1 h. Analysis of the constituent nucleosides was undertaken on an Orbitrap Exploris 240 LC–MS system without any further purification. Nucleoside composition of the PTQ dienone-treated DNA duplex was calculated (see Table S2 in the Supporting Information) from the peak areas of the respective nucleosides in the HPLC trace (Figure 3). Analysis is shown in Figure 2.

### Recognition of the *O*^6^-PTBG Adduct by MGMT and Atl1

#### MGMT Recognition of *O*^6^-PTBG: Pstl Restriction Endonuclease Assays

Reaction mixtures (20 μL) contained 10 pM MGMT protein and 50 fmol 23mer *O*^6^-alkylguanine (ODN-1 or ODN-6): 5′-HEX-labeled complement ODN-5 duplex in 50 mM Tris-HCl, pH 8.3, 3 mM DTT, and 1 mM EDTA. Reactions that were subjected to incubations were carried out at 37 °C for 1 h, first with MGMT and second with *Pst*I. Samples were analyzed by PAGE (20%) using Typoon 9200, Variable Mode Imager by Amersham Biosciences.

#### MGMT Recognition of *O*^6^-PTBG: Determination of IC_50_ Values for the *O*^6^-AlkG ODNs

The assay used the method of Watson et al.^36^ All radioactive MGMT assays were completed with ds DNA, and each incubation was completed at 37 °C for 90 min. Scintillation was measured on a Tri Carb 1900 TR Liquid Scintillation Analyzer by Packard over 5 min periods for each sample, and the IC_50_ was calculated from these values (Figure S6, Supporting Information).

#### Atl1 Recognition of *O*^6^-PTBG-Containing ODNs Using EMSA

Reaction mixtures (20 μL) contained 50 fmol 23mer *O*^6^-alkylguanine (ODN-1 or ODN-6): 5′-HEX-labeled complement oligonucleotide duplex (ODN-5) in 50 mM Tris-HCl, pH 8.3, 3 mM DTT, and 1 mM EDTA and varying concentrations of Atl1-MBP or Atl1 proteins. Reactions were incubated at 37 °C for 1 h and then analyzed by native PAGE (15%) using Typoon 9200, Variable Mode Imager by Amersham Biosciences.

## Results and Discussion

**Scheme 2:**
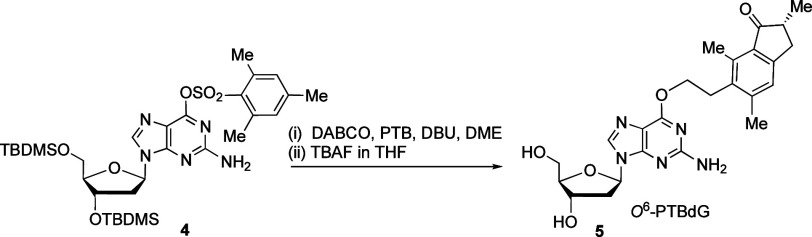
Synthesis of the *O*^6^-PTBdG Nucleoside Standard (5)

To characterize and quantify DNA damage derived from exposure to PTQ dienone, we planned to identify constituent nucleosides following enzymatic digestion. We, therefore, synthesized a nucleoside standard of *O*^6^-PTBdG (5) by following a literature procedure,^35^ reacting 3′,5′-di-*O*-TBDMS-protected 6-mesitylenesulfonyl-2′-deoxyguanosine (4) with PTB,^33^ DABCO, and DBU in DME (Scheme 2). Subsequent removal of the TBDMS protecting groups with TBAF in THF and purification by RP-HPLC followed by preparative TLC afforded nucleoside standard 5, whose structure was confirmed by MS and NMR.

To establish that complete enzymatic digestion of DNA containing *O*^6^-PTBdG (5) to constituent nucleosides could be achieved, we prepared a 23mer oligodeoxyribonucleotide (ODN-1) (5′-GAA CT(*O*^6^-PTBG) CAG CTC CGT GCT GGC CC). Thus, an ODN precursor containing the convertible base 2-amino-6-methylsulfonylpurine^31^ was reacted with PTB^33^ in DBU/acetonitrile. Following deprotection with aqueous ammonia, desalting, and purification by RP-HPLC, MS analysis revealed that the ODN mass was 40 Da higher than predicted. This suggested that an acrylonitrile adduct formed during the removal of the cyanoethyl protecting groups. In a repeated synthesis, the desired ODN was obtained successfully by including an initial brief treatment of the support-bound ODN with DBU/acetonitrile prior to the displacement reaction. ESI MS confirmed the correct mass of 7201.6 for ODN-1 (Figure 2). Enzymatic digestion of ODN-1 to constituent nucleosides followed by analysis using RP-HPLC confirmed the presence of the modified nucleoside *O*^6^-PTB in the expected ratio with the other nucleosides (Figure 2).

PTQ dienone was prepared from PTQ following the previous literature.^12^ Thus, PTQ was subject to a brief treatment with 0.02 M aq sodium carbonate before extraction into 2:1 hexane/diethyl ether. Formation of PTQ dienone was confirmed by RP-HPLC and ESI-MS spectra, which revealed two products of PTQ decomposition: PTQ dienone as a later-eluting, less polar product together with PTB derived from partial hydrolysis (Figure S1). A 30-mer DNA duplex ODN-3/ODN-4 (100 nmol) was then treated with approximately 2 mg of PTQ dienone at 37 °C for 2 h. The DNA was then desalted and subjected to enzymatic digestion, and the mixture was analyzed by LC–MS (Figure 3). This revealed a deoxyribonucleoside with a retention time identical to that of the *O*^6^-PTBdG standard (5) and the expected mass.

Further confirmation was made by comparison of MS/MS spectra obtained for the *O*^6^-PTBdG standard and corresponding species in the enzymatic digest (Figure 3D,G, respectively). This to the best of our knowledge is the first identification of the formation of an *O*^6^-PTB adduct in DNA following treatment with PTQ dienone. An earlier eluting peak (approximately 18 min) with the same *m*/*z* value as that of *O*^6^-PTBdG was also identified in the HPLC chromatogram of the nucleoside digest (Figure 3A). Using standards of *O*^6^-methyldG (*O*^6^-MedG) and *N*^7^-methyldG (*N*^7^-MedG), we ascertained that the latter nucleoside eluted significantly earlier under the same HPLC conditions (Figure S3). We, therefore, assigned the earlier eluting PTBdG nucleoside observed following HPLC analysis of the nucleoside digest to the *N*^7^-adduct. Although this component might derive from alkylation at another site on guanine such as N2, such adducts have not been identified in previous studies that have explored the reactivity of PTQ and its dienone with nucleosides and nucleic acids.^12,20^ Furthermore, products derived from N2 alkylation would be expected to have similar LC characteristics to *O*^6^-PTBdG, unlike charged more polar N7-adducts, which would elute significantly earlier by RP-HPLC, as we observe here. Using the known extinction coefficients,^12^ we calculated (Table S2) the relative amounts of *O*^6^-PTBdG and *N*^7^-PTBdG formed following treatment of the DNA duplex with PTQ dienone (Table 1). The ratio of O6/N7 alkylation observed is approximately 1.4 and similar to values previously determined in reactions of hard electrophiles with guanine in DNA.^28,30^

**Table 1:** Amount of Guanine Adducts after Treatment of Duplex DNA with PTQ Dienone

nucleoside	area^a^	corrected area	% detected
*O*^6^-PTBdG	1.00	0.25	0.7
*N*^7^-PTBdG	0.80	0.18	0.51
dG	55.30	34.56	98.57

a Calculated from HPLC chromatogram Figure 3A. See the Supporting Information (Table S2).

**Figure 4:**
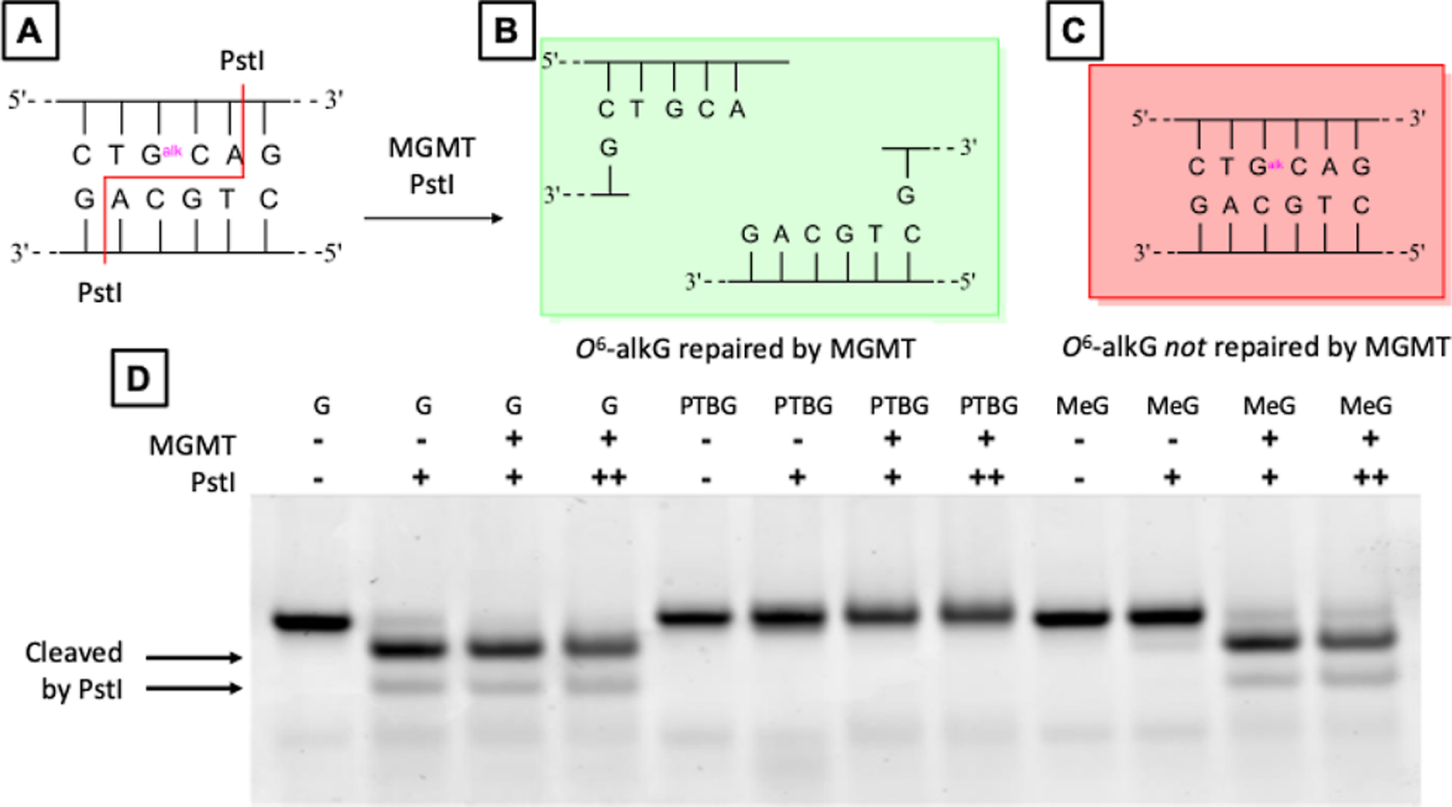
MGMT shows lack of repair of *O*^6^-PTBG: (A) *Pst*I recognition sequence containing *O*^6^-alkG in the ODN duplex; (B) MGMT repair (alkyl transfer) results in an unmodified sequence that is cleaved by *Pst*I; (C) *Pst*I does not hydrolyze duplex DNA containing *O*^6^-alkG in its recognition sequence; and (D) analysis of ODN duplexes by 20% PAGE following treatment with MGMT and *Pst*I: G represents unmodified duplex, PTBG indicates the ODN duplex containing *O*^6^-PTBG, Me indicates the ODN duplex containing *O*^6^-MeG.

Although *O*^6^-alkGs are known to be toxic, mutagenic, and carcinogenic, their ability to induce such effects can be determined by how efficiently they are repaired by MGMT.^26^ Previously, we and others have shown that certain *O*^6^-alkGs such as 2-hydroxyethyl^31^ and 4-(3-pyridyl)-4-oxobutyl^32^ are rather poor substrates for MGMT. To assess the ability of MGMT to repair *O*^6^-PTBG, we annealed ODN-1 to an unmodified complementary duplex, ODN-5, creating a *Pst*I restriction endonuclease recognition sequence encompassing the adduct. A duplex containing *O*^6^-MeG in the *Pst*I site was resistant to cleavage by *Pst*I, but following incubation with MGMT and then *Pst*I, complete cleavage of the duplex was observed (Figure 4). In contrast, when the *O*^6^-PTBG-containing sequence was treated in the same way, no digestion of the DNA was observed, suggesting that the adduct is not a substrate for MGMT under the assay conditions used.

Further assessment of the ability of MGMT to repair *O*^6^-PTBG was obtained using a radioisotope-based competition assay,^36^ in which MGMT is first incubated with lesion-containing ODNs and residual activity if quantified after subsequent incubation with DNA containing tritiated *O*^6^-MeG. In this assay, the IC_50_ (ODN concentration resulting in 50% inactivation of MGMT) for the *O*^6^-MeG and *O*^6^-PTBG duplexes was ∼2 nM and ∼200 nM, respectively (Figure S11). This indicates that *O*^6^-PTBG is a poor MGMT substrate relative to *O*^6^-MeG, possibly resulting in the accumulation of this damage with prolonged exposures. In this context, earlier studies have shown that MGMT and the *Escherichia coli* alkyltransferase, AGT, repaired a series of *O*^6^-alkGs in DNA in vitro at different rates, but these were generally slower as the size and complexity of the alkyl group increased.^37^ In various human and Chinese hamster cell lines, repair of higher *O*^6^-alkGs in DNA has been reported to be dependent upon NER^38,39^ or both MGMT and NER.^39,40^ The potential genotoxicity of a poor substrate for MGMT will thus depend on how well it is recognized and processed by other repair systems in any particular organism. Indeed, the antitumor drug, Illudin S, is structurally related to PTQ, and the damage it introduces into DNA is processed by transcription- or replication-coupled NER.^41^

**Figure 5:**
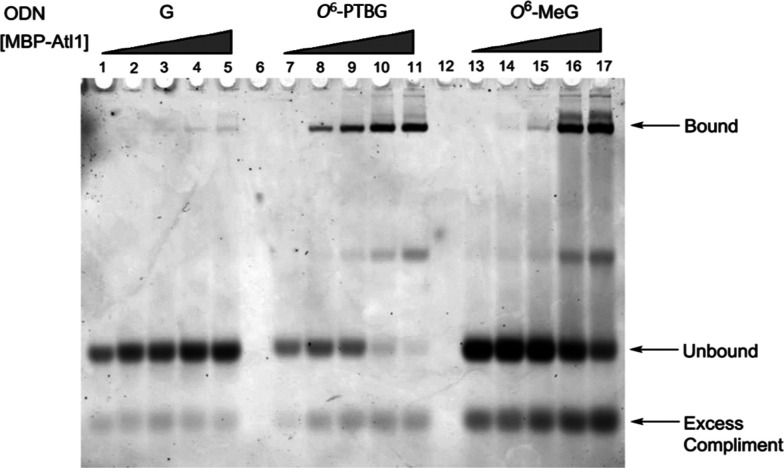
EMSA of ds 23mer ODNs following incubation with increasing concentrations of MBP-Atl1. Duplexes were of unmodified guanine (G; ODNs 2 + 5 lanes 1–5), *O*^6^-PTBG (ODNs 1 + 5 lanes 7–11), and *O*^6^-MeG (ODNs 5 + 6). Lanes 13–17. The migration positions of the excess complement, unbound duplex, and bound duplex are shown.

We also investigated the *Schizosaccharomyces pombe* protein Atl1, which recognizes *O*^6^-alkGs in ODNs and binds with high affinity but, unlike MGMT, does not transfer the alkyl group.^42^ As shown in Figure 5, titrating Atl1 protein with ODNs that contain either *O*^6^-PTBG or *O*^6^-MeG results in a concentration-dependent formation of a protein–DNA complex, seen as a slower-running band on native PAGE. Very little binding to the G control duplex was seen, and with the *O*^6^-PTBG ODN, almost all the duplex was shifted, whereas some residual unbound ODN was seen with the *O*^6^-MeG duplex, indicating stronger binding to the former than to the latter. This confirms that *O*^6^-PTBG is recognized and bound by Atl1, in common with ODNs containing all other *O*^6^-alkGs that we have previously tested.^43^

The exposure of DNA to PTQ dienone derived from the bracken carcinogen PTQ results in the formation of a variety of DNA adducts. Here, we have synthesized an ODN containing the *O*^6^-guanine adduct derived from this reaction, namely, *O*^6^-PTBG, and we have shown for the first time that exposure of DNA to PTQ dienone results in the formation of this adduct. In common with other *O*^6^-alkGs, *O*^6^-PTBG is likely to be mutagenic and possibly toxic. Furthermore, since it is very poorly repaired by MGMT, it may be persistent and more deleterious. The carcinogenicity of bracken and PTQ could, therefore, be attributed to the formation of *O*^6^-PTBG in DNA.

## Supporting Information Available

Supporting figures, MS spectra of oligodeoxyribonucleotides, and NMR spectra of compounds (PDF)

## Abbreviations

ESI MS: electrospray ionization mass spectrometry
MGMT: *O*^6^-methylguanine-DNA-methyltransferase
*N*^3^-alkA: *N*^3^-alkyladenine
*N*^7^-alkG: *N*^7^-alkylguanine
*O*^6^-alkG: *O*^6^-alkylguanine
ODN: oligodeoxyribonucleotide
PTB: pterosinB
PTQ: ptaquiloside
RP-HPLC: reversed phase high-performance liquid chromatography
TBDMS: *tert*-butyldimethylsilyl
TEAA: triethylammonium acetate
