# Supplementary material for: Bracken Fern Carcinogen, Ptaquiloside, Forms a Guanine O6-Adduct in DNA
Source: J Agric Food Chem. 2025 Jan 7;73(2):1053–61. doi: 10.1021/acs.jafc.4c07187 (PMC11741102; doi:10.1021/acs.jafc.4c07187)
Supplement: Supplementary file 1 — jf4c07187_si_001.pdf [file jf4c07187_si_001.pdf]

The Bracken Fern Carcinogen, Ptaquiloside, Forms a Guanine O^6-Adduct in DNA

Fourat Keskin, Hannah Noone, Mark J. Dickman, Esther Allen, William D. Mulcrone, Lars Holm Rasmussen, Hans Christian Bruun Hansen, Peter J. O'Connor, Andrew C. Povey, Geoffrey P. Margison, and David M. Williams*

Electronic Supporting Information

Table of Contents

1. Nucleoside composition analysis of ODN-1 Page 3

2. Reaction of ODN duplex with PTQ dienone Page 4

3. HPLC traces Page 7

4. UV-Vis spectra Page 8

5. MGMT recognition of O6-PTBG Page 9

6. 1H and 13C NMR spectra Page 10

7. ESI MS data Page 21

8. References Page 25

1. Nucleoside composition analysis of ODN-1

The HPLC trace is shown in Figure 3A in main text. Analytical RP-HPLC used a Phenomenex Gemini C18 5 µm 4.6 x 250 mm column, at 40°C and a flow rate of 1 mL/min and gradient of 3-75% MeCN:H_2O over 36 mins. with absorbance measured at 260 nm. Nucleoside composition of ODN-1 was calculated from the peak areas of the respective nucleosides in the HPLC trace. Analysis is shown in Table S1 below.

Quantification of 23-Mer O6-PTBdG ODN digest

Nucleoside	Integrated Area	Cofactor	Corrected Area (Area/cofactor)	Corrected Area %	# of bases this equates to	# of bases in ODN
**dC**	170,285	1.00	170,285	40.8	9.4	9
**dG**	187,954	1.60	117,270	28.1	6.5	6
**T**	84,086	1.21	69,753	16.7	3.8	4
**dA**	106,608	2.11	50,535	12.1	2.8	3
**O6-PTBdG**	40,267	4.16	9,668	2.3	0.6	1
**Total**			417,512	100.0	23.0	23

Nucleoside	Ext. coefficient	Cofactor
**dC**	7,300	1.00
**dG**	11,700	1.60
**T**	8,800	1.21
**dA**	15,400	2.11
**O6-PTBdG**	30404	4.16

Nucleoside	Literature ε[1] (252 nm)	Absorbance of O6-PTBdG at 260 nm	Absorbance of O6-PTBdG at 252 nm	Correction factor	Corrected ε (260 nm)
**O6-PTBdG**	29100	0.21618	0.20691	1.04480208	30404

1. Reference: Ojika et al, Tetrahedron 1987

Table S1. Nucleoside composition analysis for ODN-1: The extinction coefficients at 260nm for the natural nucleosides and *O*^{6}-PTBdG are shown in the table. The extinction coefficient for *O*^{6}-PTBdG was corrected from the literature value at 252nm using an absorption spectrum of *O*^{6}-PTBdG (compound 5). These were divided by the extinction coefficient of dC (the lowest molar extinction coefficient) to provide a cofactor. The integrated areas of the peaks were then divided by this cofactor to give a corrected area, which was then converted to a % of the total peak areas and multiplied by 23 to give the number of nucleosides present.

2. Reaction of ODN duplex with PTQ dienone

Formation of PTQ dienone (described in main text) was confirmed by HPLC and MS (see Figure S1 below). RP-HPLC using a Phenomenex Gemini C18 5 μm 4.6 x 250 mm column, at 40°C and a flow rate of 1 mL/min and a gradient of 3-80% MeCN:H_2O over 40 mins with absorbance measured at 260 nm.

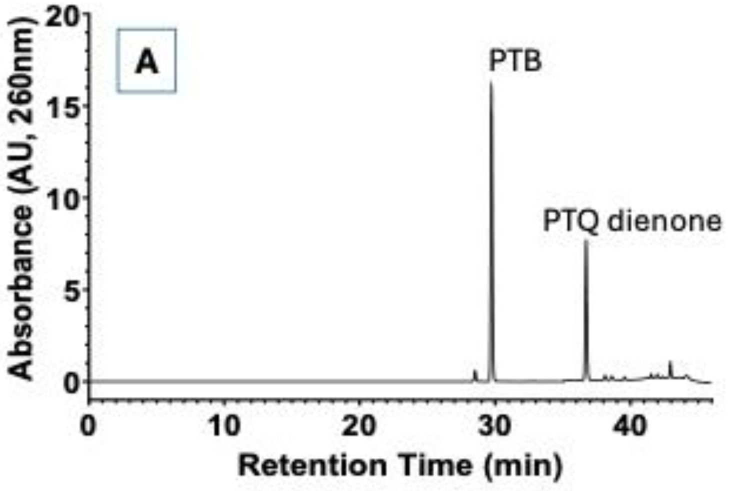

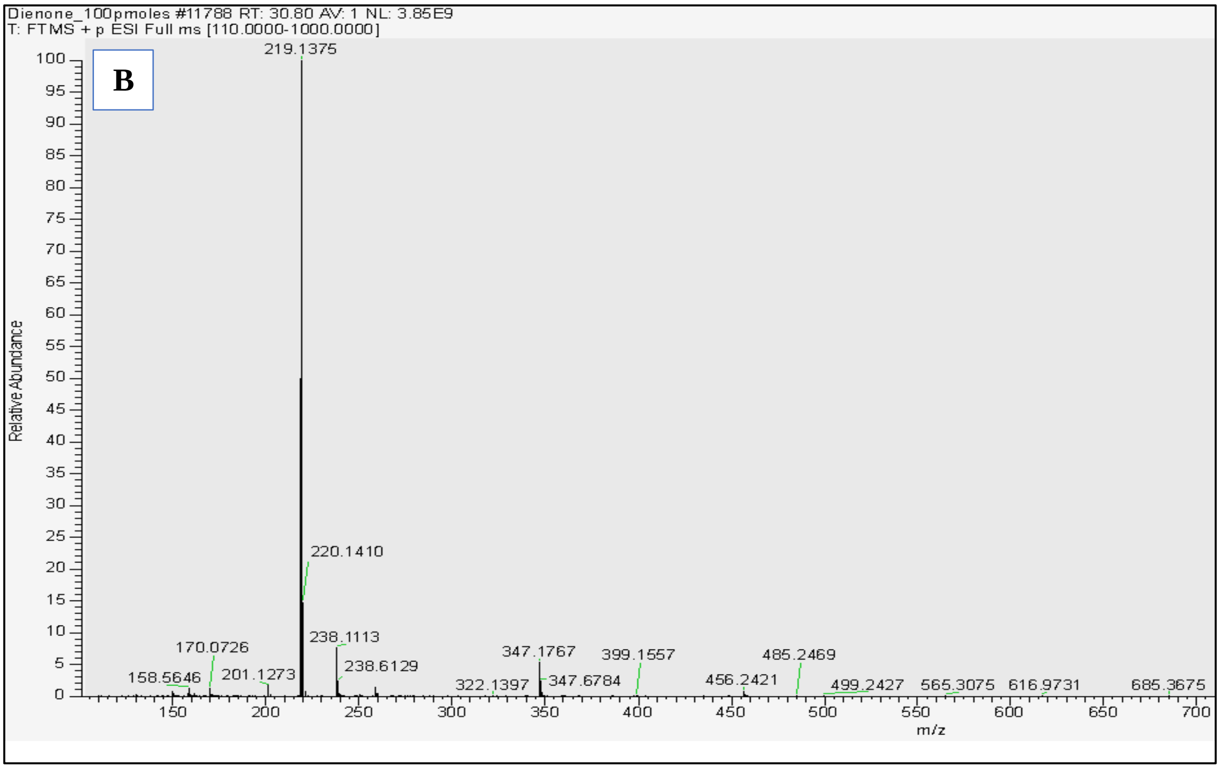

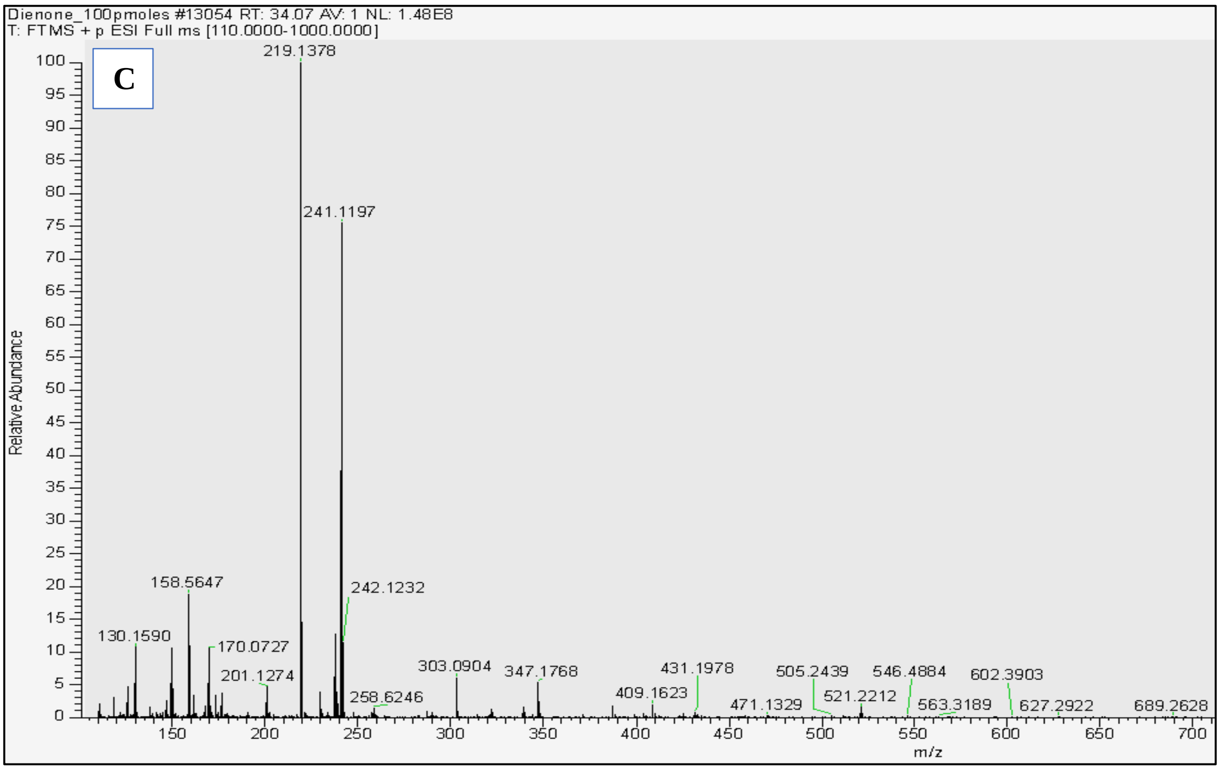

Figure S1: RP-HPLC chromatogram of PTB and PTQ dienone formed from treatment of PTQ with aq Na_2CO_3 and respective ESI-MS spectra. **A** RP-HPLC chromatogram of PTB and PTQ dienone products at 29.8 and 36.8 min, respectively. RP-HPLC used a Phenomenex Gemini C18 5 µm 4.6 x 250 mm column, at 40°C and a flow rate of 1 mL/min and a gradient of 3-80% MeCN:H_2O over 40 mins. with absorbance measured at 260 nm.; **B** ESI-MS spectrum of PTB showing $[M+H]^+$ peak; **C** ESI-MS spectrum of PTQ dienone showing $[M+H]^+$ peak at and $[M+Na]^+$.

Reaction of PTQ dienone with duplex DNA

Reaction of PTQ dienone with DNA followed by nucleoside digestion analysis is described in the main text. Nucleoside composition of PTQ dienone-treated DNA duplex was calculated from the peak areas of the respective nucleosides in the HPLC trace (Figure 5, main text). Analysis is shown in Table S2 below. HPLC conditions are described in section 1 above

Quantification of DNA damage following digest of PTQ dienone treated DNA

Nucleoside	Retention time	Integrated Area	Cofactor	Corrected Area (Area/cofactor)	Corrected Area %	# of bases this equates to	# of bases in ODN
dC	3.5	25.60	1.00	25.60	21.63	12.98	13.0
dG	12.9	55.30	1.60	34.56	29.20	17.52	17.0
dT	14	30.70	1.21	25.37	21.43	12.86	13.0
dA	12.8	68.40	2.11	32.42	27.39	16.43	17.0
O6-PTBdG	29.5	1.00	4.16	0.24	0.20	0.12	
N7-PTBdG	18.2	0.80	4.52	0.18	0.15	0.09	
Total				118.4	100	60.0	60

Nucleoside	Extinction coefficient	Cofactor
dC	7,300	1.00
dG	11,700	1.60
T	8,800	1.21
dA	15,400	2.11
O6-PTBdG	30404	4.16
N7-PTBdG	33,000	4.52

Total dG area	35.0
% of dG O^6-alkylated	0.69
% of dG N^7-alkylated	0.51
% unreacted dG	98.81
Ratio O^6/N^7	1.4

Nucleoside	Literature[1] ε (wavelength)	Absorbance at 260 nm of PTB	Absorbance at (nm)of PTB	Correction factor	Corrected ε
O6-PTBdG	29100 (252 nm)	0.21618	0.20691	1.044802088	30404
N7-PTBdG	33000 (258 nm)				

1. Reference: Ojika et al, Tetrahedron 1987

Table S2. Nucleoside composition analysis for PTQ dienone-treated DNA duplex: The extinction coefficients at 260nm for the natural nucleosides and O^6-PTBdG and N^7-PTBdG are shown in the table. The extinction coefficient for O^6-PTBdG was corrected to 260nm from the literature value[1] using the absorption spectrum of the respective nucleoside O^6-PTBdG (see Figure S4). Extinction coefficients for O^6-PTBdG and N^7-PTBdG were divided by the extinction coefficient of dC (the lowest molar extinction coefficient) to provide a cofactor. The integrated areas of the peaks were then divided by this cofactor to give a corrected area, which was then converted to a % of the total peak areas and multiplied by 60 to give the number of nucleosides present.

3. HPLC Traces of nucleoside analogues

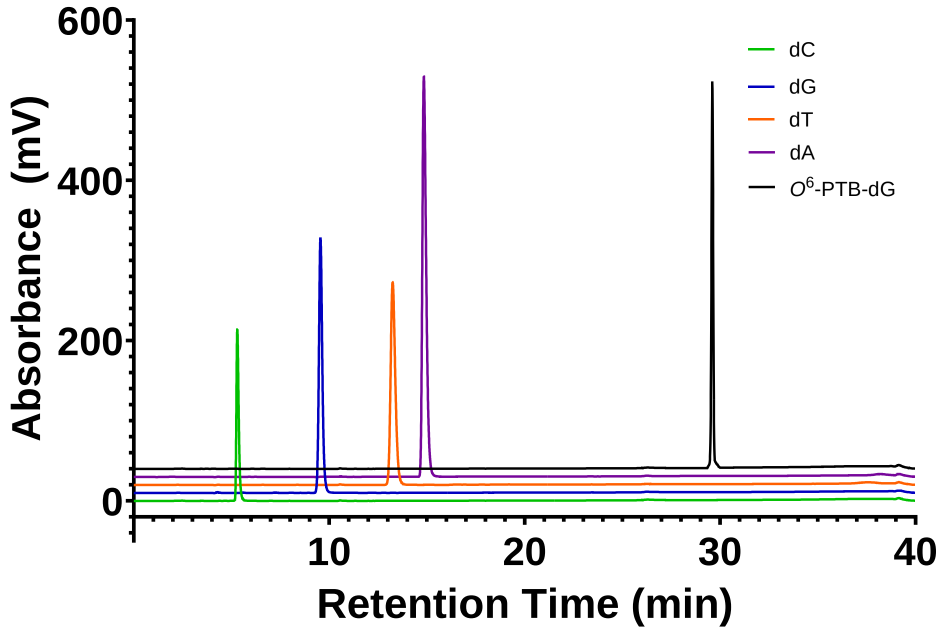

Figure S2: HPLC trace of canonical nucleosides and *O*[6]-PTBdG. The gradient used was 3-75% MeCN:H_2O over 36 mins using the conditions shown in section 1 above

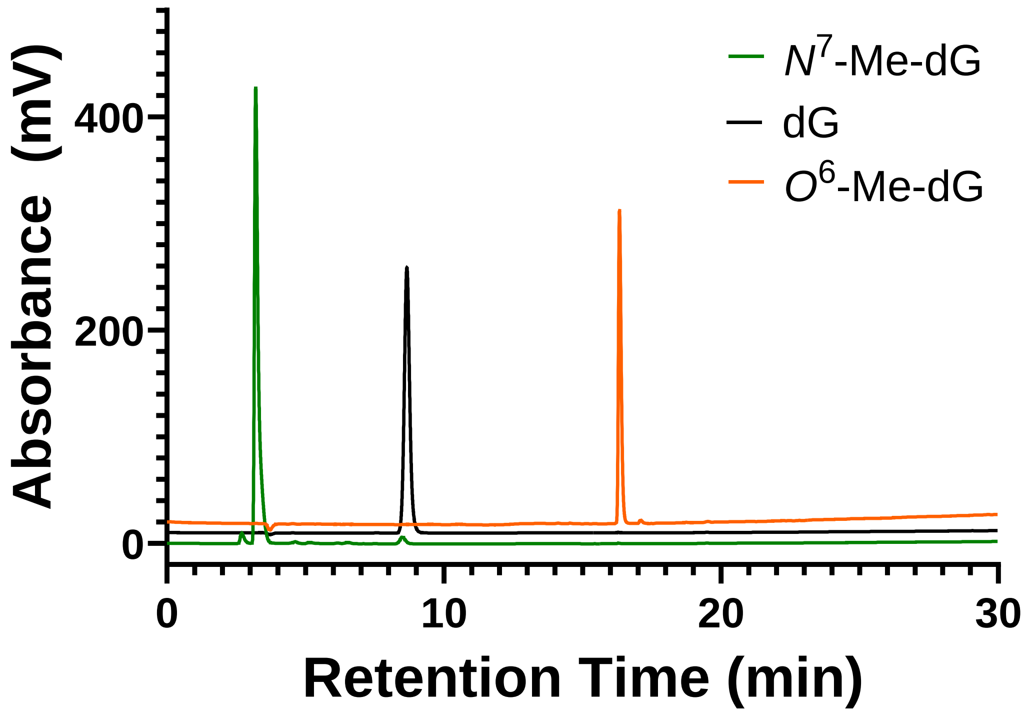

Figure S3: HPLC trace of dG, *N*[7]-MedG and *O*[6]-MedG. The gradient used was 3-75% MeCN:H_2O over 36 mins. using the conditions shown in section 1 above.

4. UV-Vis Spectra

***O*^6-PTBdG**

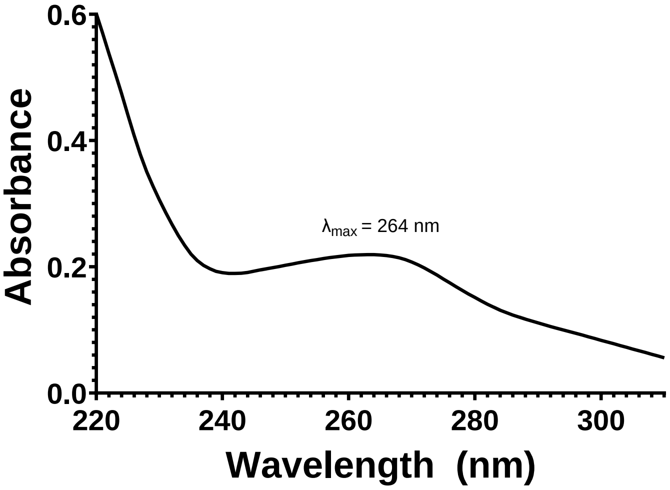

Figure S4: UV-Vis trace of O^6-PTBdG from 220-310 nm in MeOH.

dG, *O*^6-MedG, *N*^7-MedG

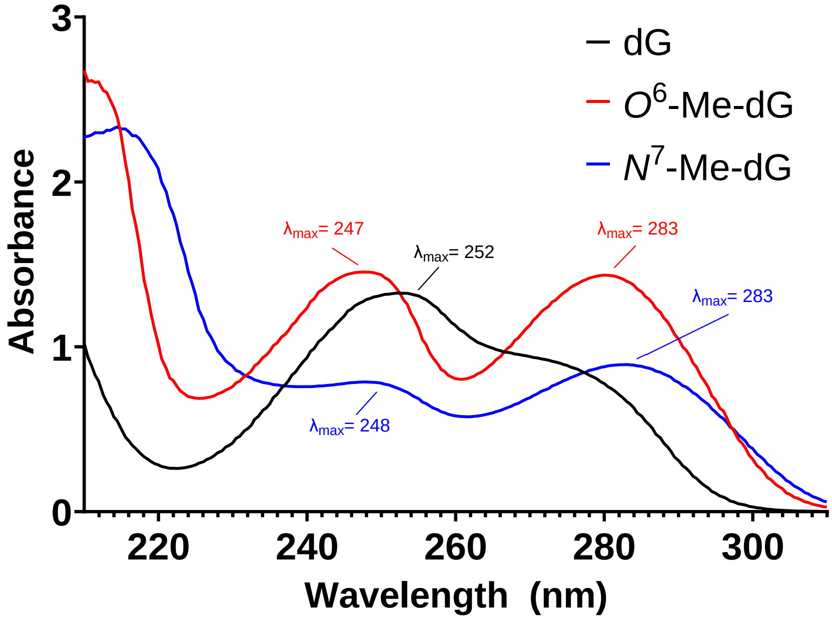

Figure S5: UV-Vis trace of dG, *O*^{6}-MedG, and *N*^{7}-MedG from 210-310 nm in H_2O.

5. MGMT recognition of O6-PTBG: Determination of IC50 values for O6-alkG ODNs.

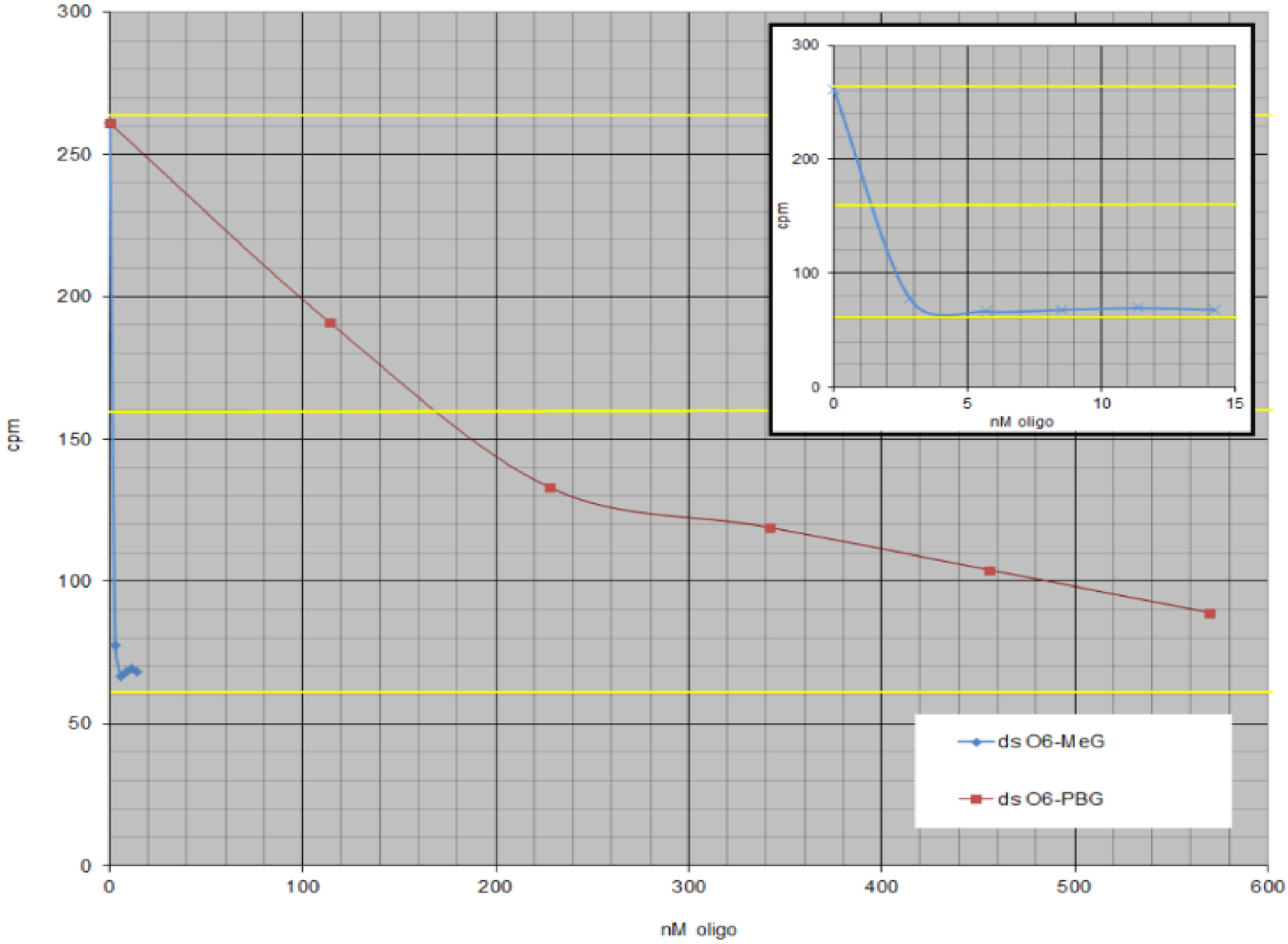

Figure S6: Dose response curves for inactivation of MGMT with ds O^6-MeG, and ds O^6-PTBG. Yellow lines show maximum recorded radioactivity, mean and minimum recorded radioactivity (cpm) used to calculate IC50 concentrations for O^6-PTBG (red) and ds O^6-MelG (blue). The expansion shows O^6-MeG from 0 to 15 nM of oligo.

6. ^{1}H and 13C NMR spectra

3′,5′-Bis-*O*-(*t*-butyldimethylsilyl)-2′-deoxyguanosine (^{1}H NMR in d6 DMSO)

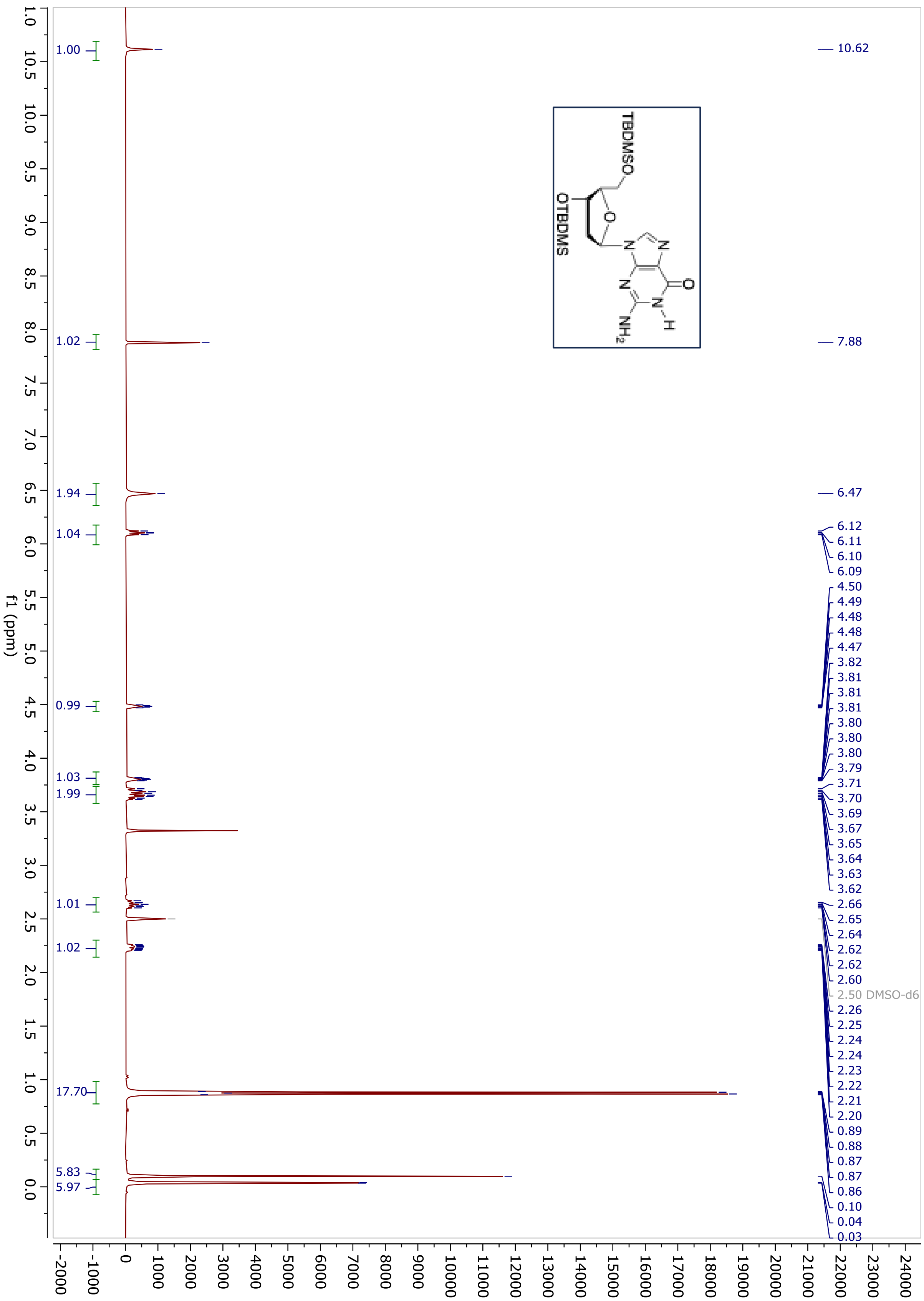

3′,5′-*Bis*-*O*-(*t*-butyldimethylsilyl)-2′-deoxyguanosine (^{13}C NMR in d6 DMSO)

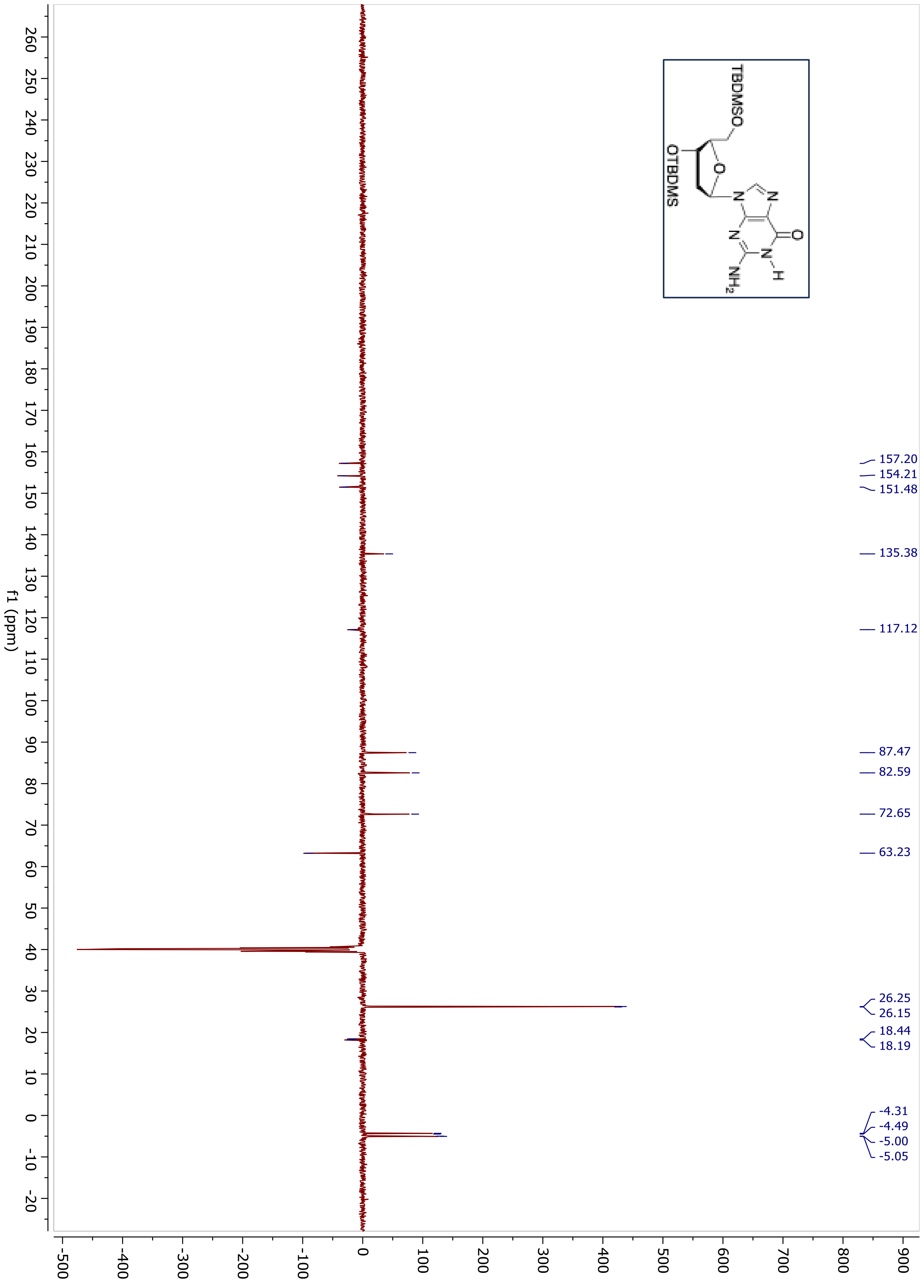

O^6-Mesitylenesulfonyl-3′,5′-*bis*-*O*-(*t*-butyldimethylsilyl)-2′-deoxyguanosine

(^{1}H NMR in d6 DMSO)

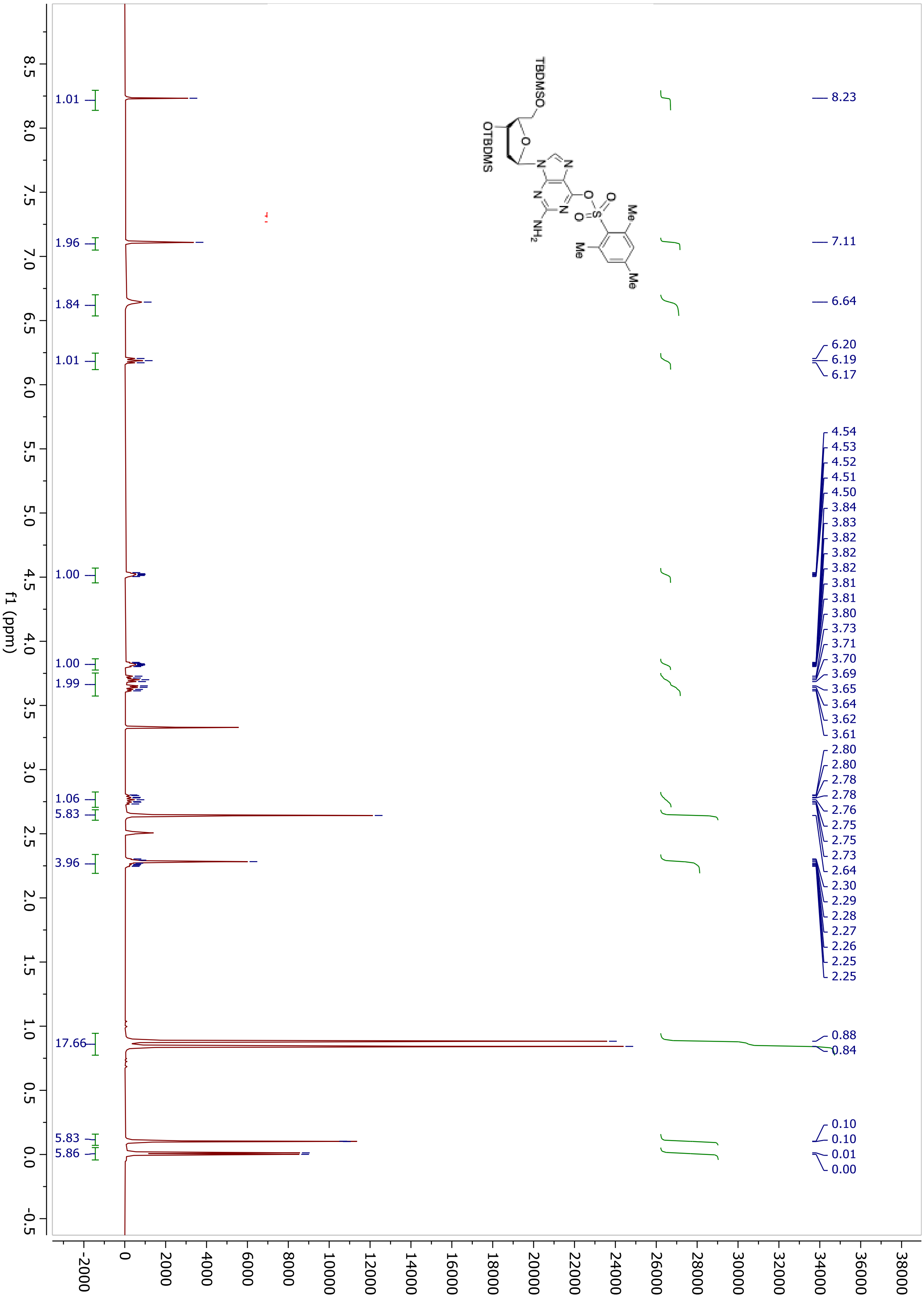

O^6-Mesitylenesulfonyl-3′,5′-*bis*-*O*-(*t*-butyldimethylsilyl)-2′-deoxyguanosine

(^{13}C NMR in d6 DMSO)

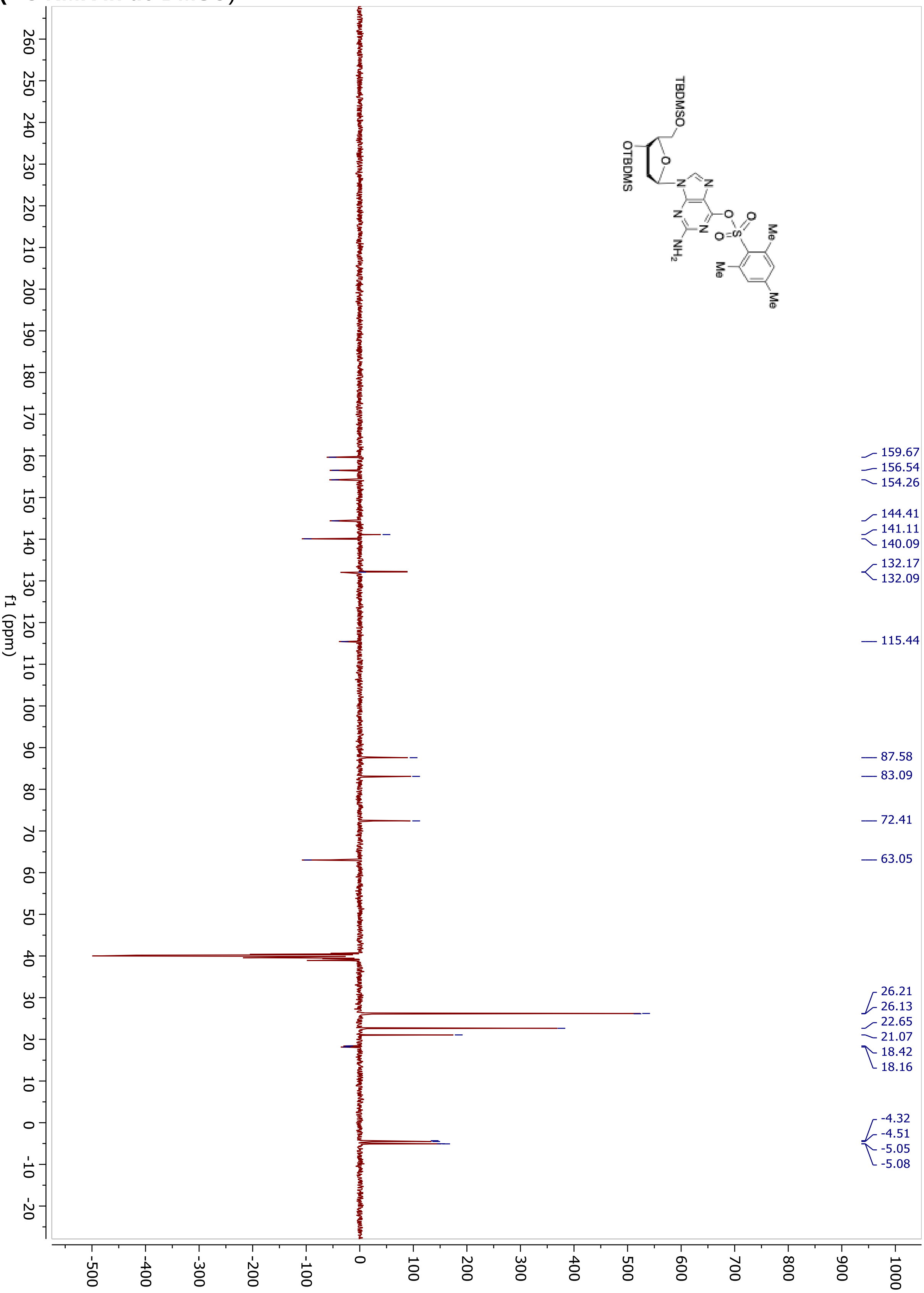

O[6]-PTB-2′-deoxyguanosine (^{1}H NMR in d6 DMSO)

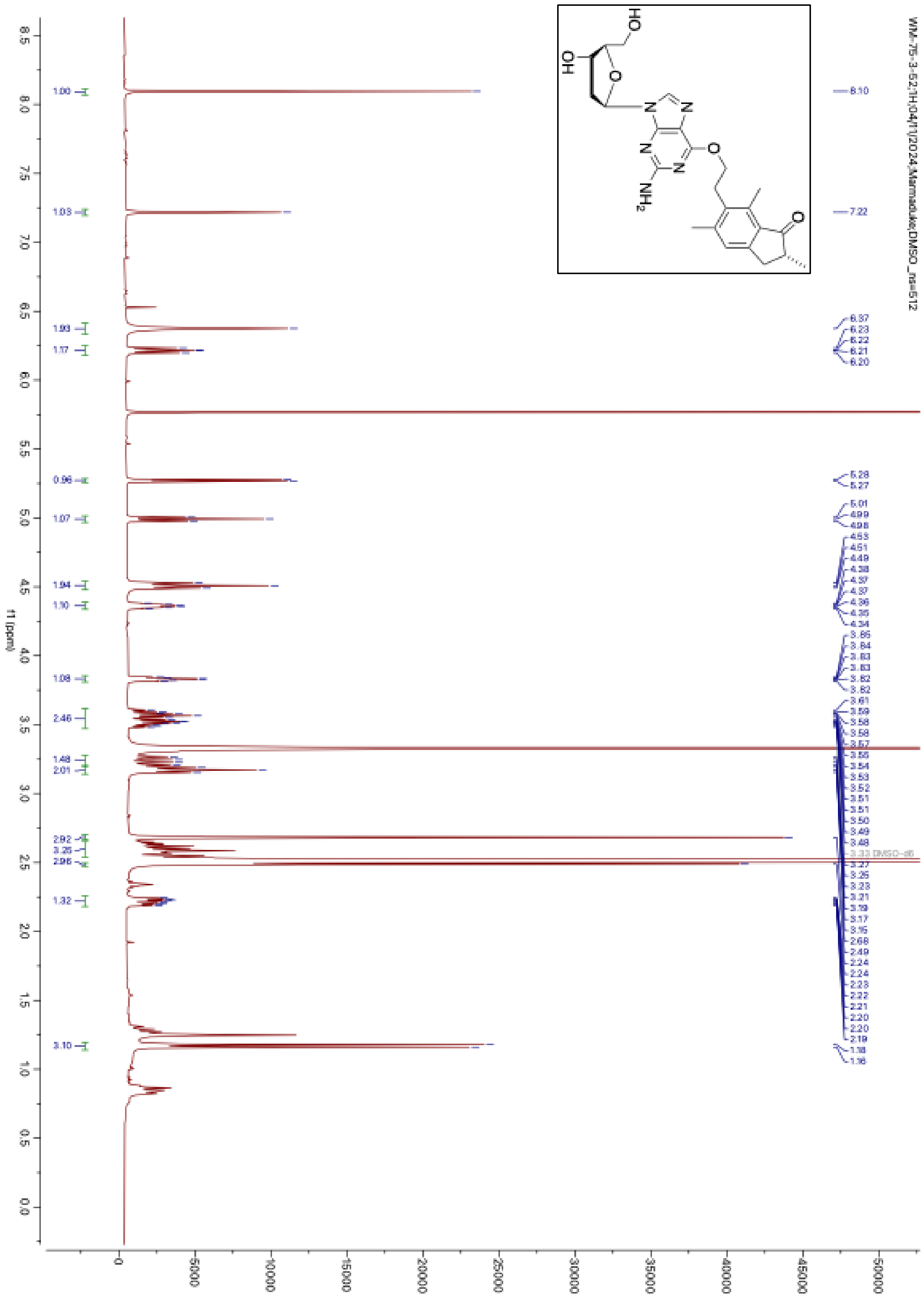

O^6-PTB-2′-deoxyguanosine (^{13}C NMR in d6 DMSO)

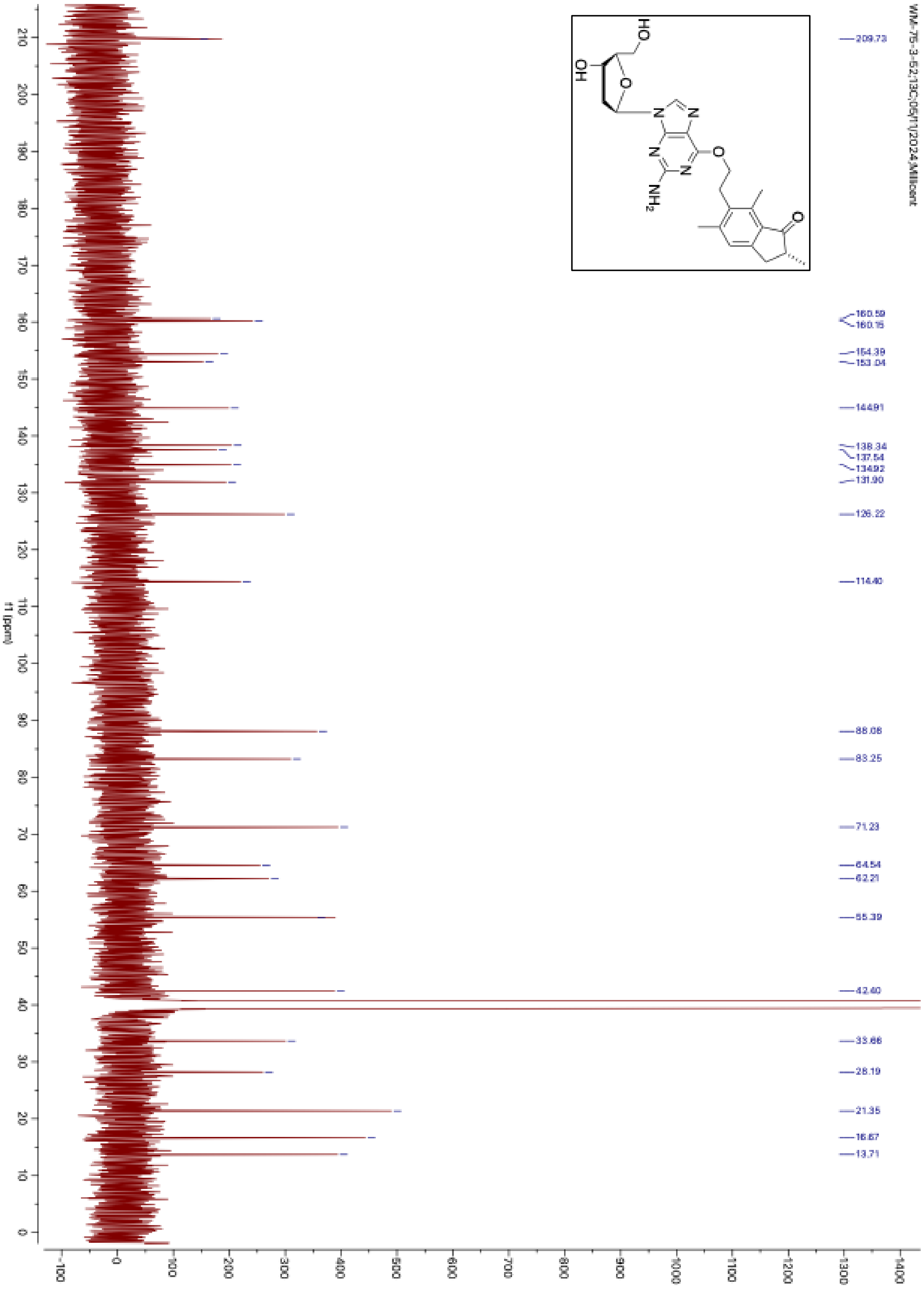

O[6]-Methyl-2′-deoxyguanosine (^{1}H NMR in d6 DMSO)

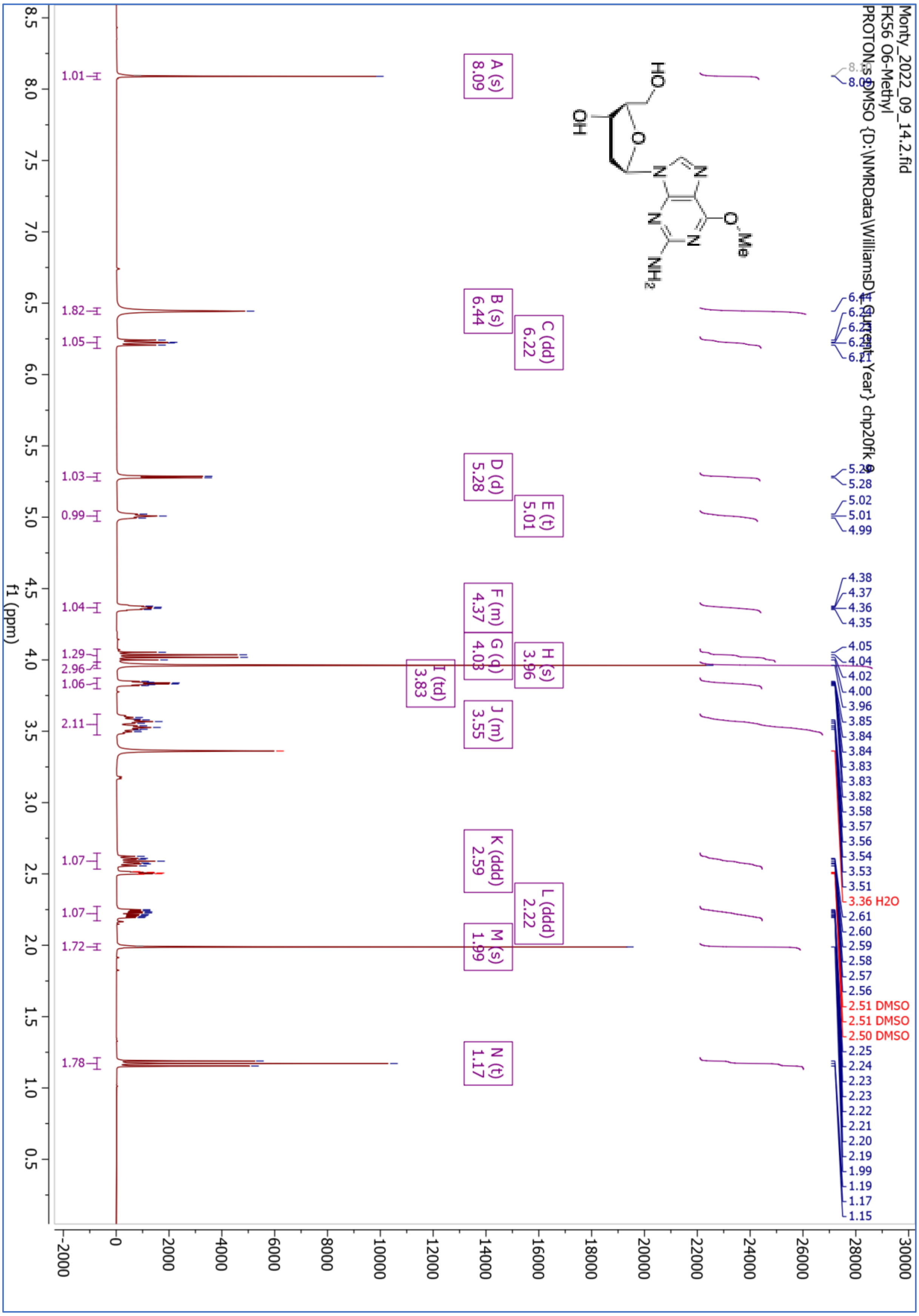

O^6-Methyl-2′-deoxyguanosine (^{13}C NMR in d6 DMSO)

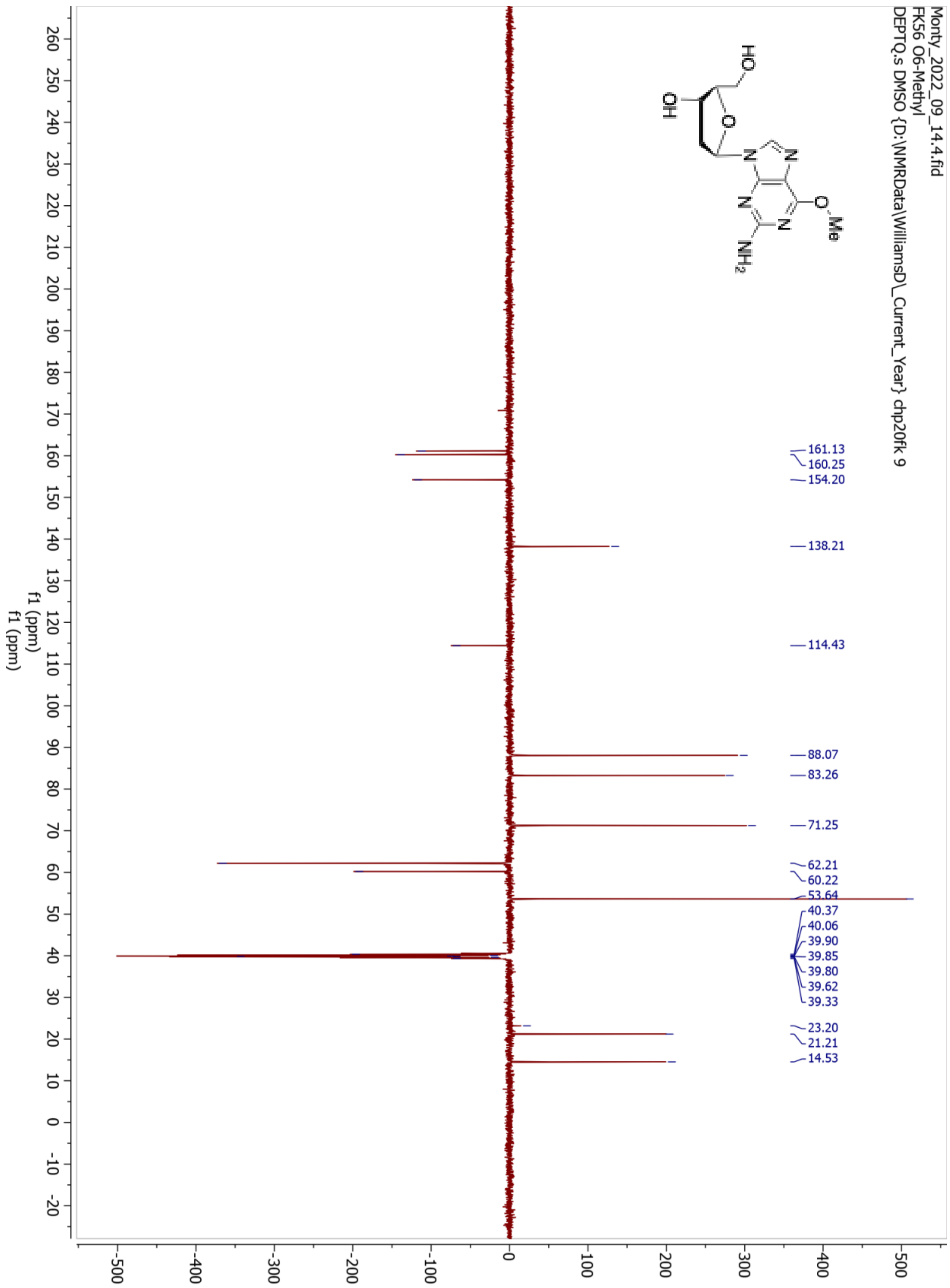

***N*[7]-Methyl-2′-deoxyguanosine (^{1}H NMR in d6 DMSO)**

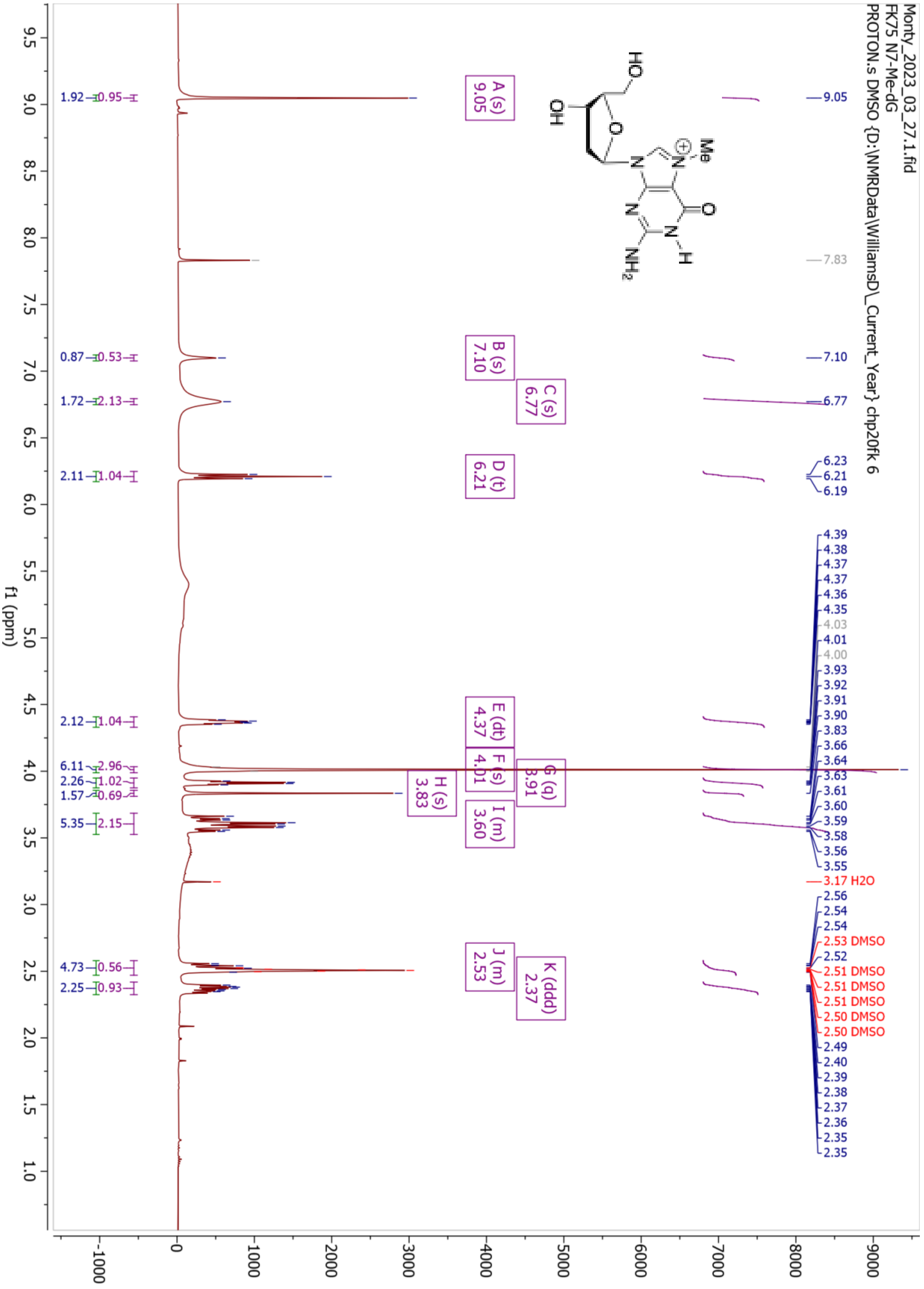

N^7-Methyl-2′-deoxyguanosine (^{13}C NMR in d6 DMSO)

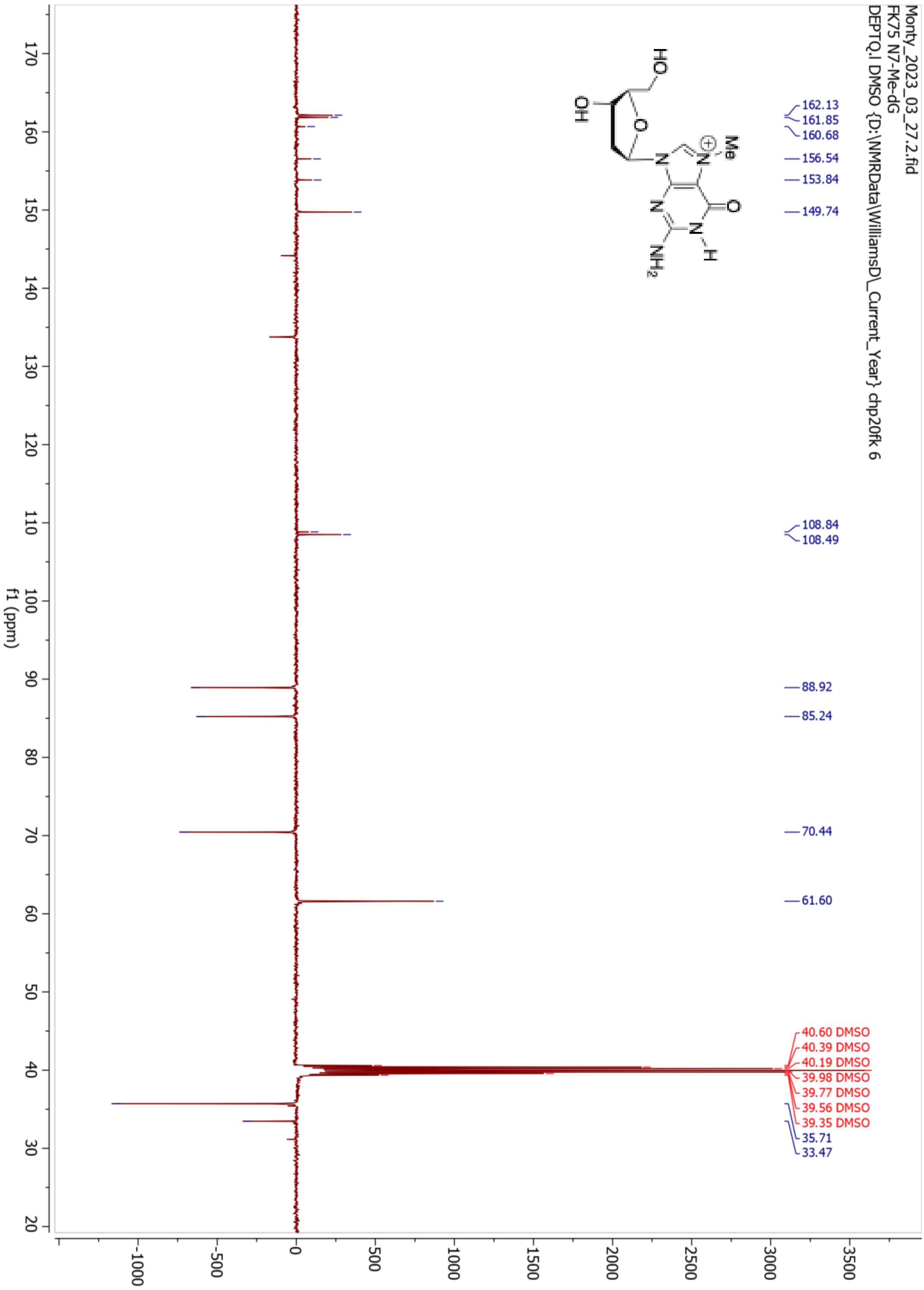

7. ESI Mass Spectra

3′,5′-*Bis*-*O*-(*t*-butyldimethylsilyl)-2′-deoxyguanosine

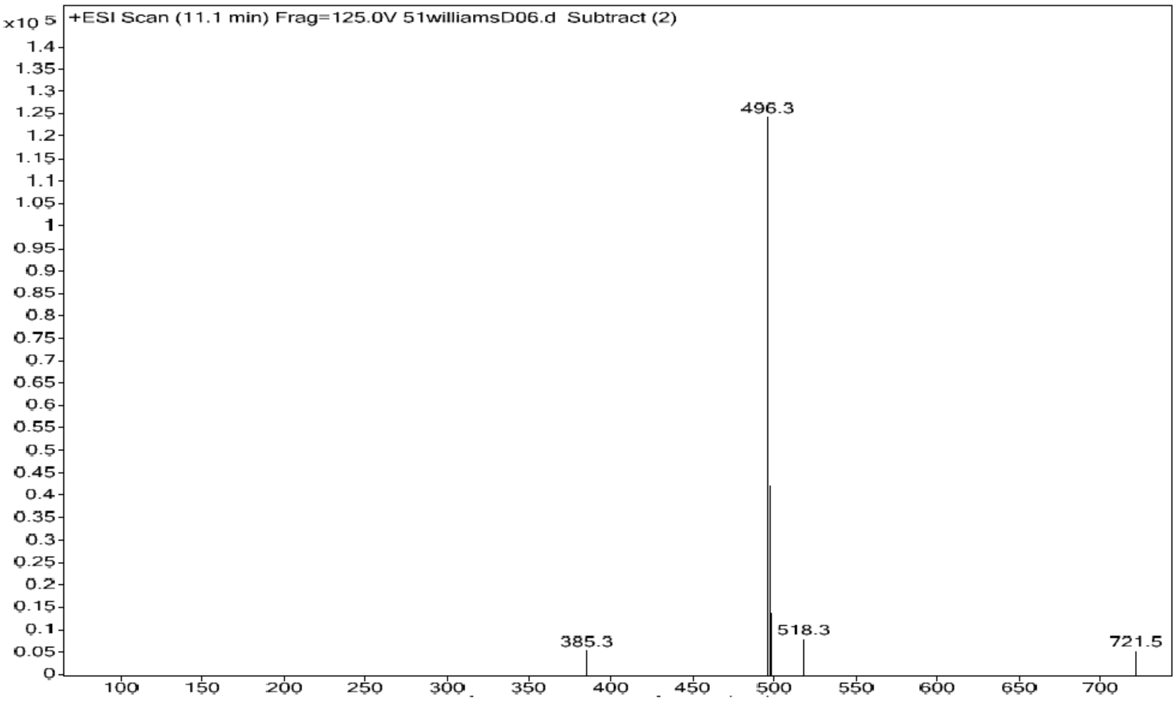

User Spectra

Fragmentor Voltage	Collision Energy	Ionization Mode
125	0	ESI

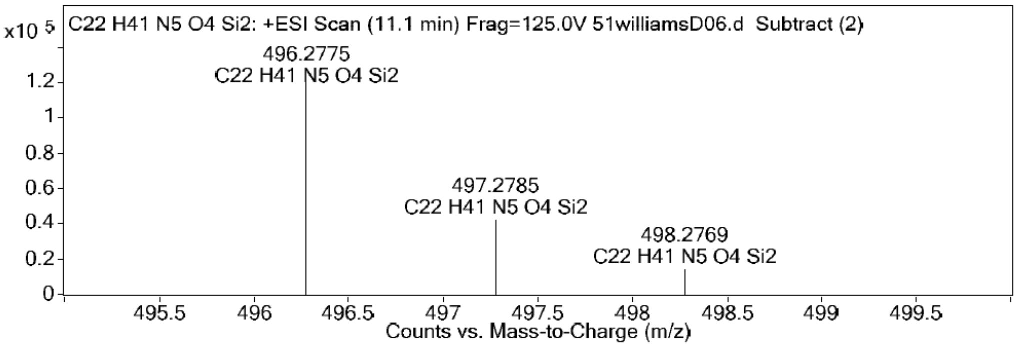

Peak List

m/z	z	Abund	Formula	Ion	PPM Difference	Calculated m/z
496.2775	1	124127.28	C22 H41 N5 O4 Si2	(M+H)+	-1.12	496.277

O^6-Mesitylenesulfonyl-3′,5′-*bis*-*O*-(*t*-butyldimethylsilyl)-2′-deoxyguanosine

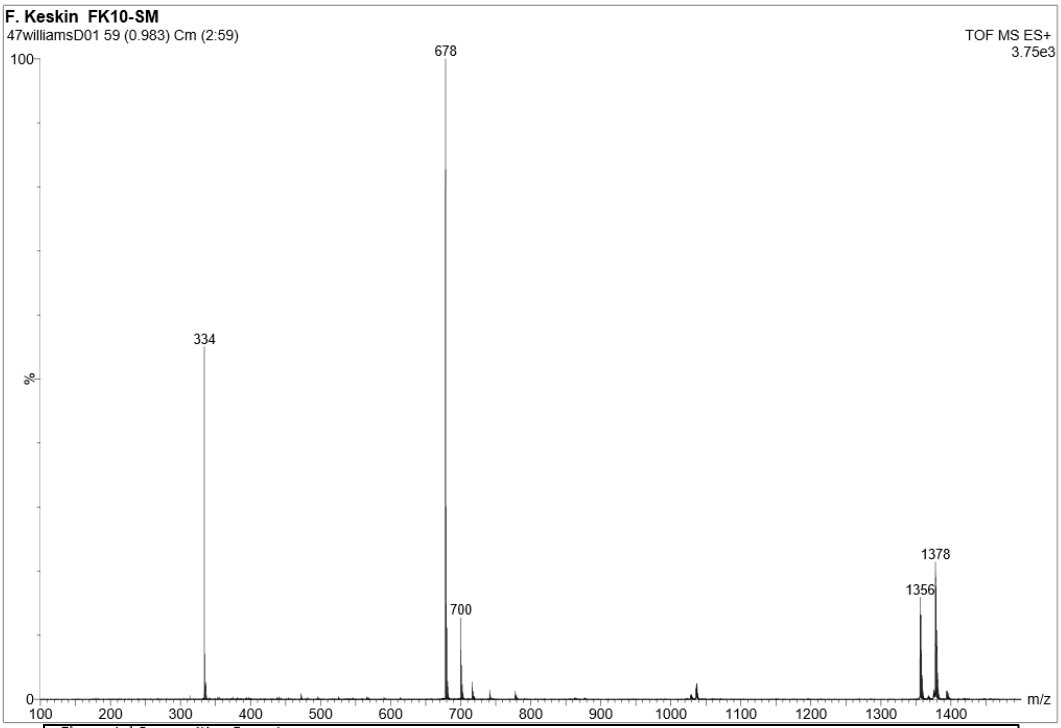

Elemental Composition Report

Single Mass Analysis

Tolerance = 5.0 PPM / DBE: min = -1.5, max = 50.0

Element prediction: Off

Number of isotope peaks used for i-FIT = 3

Monoisotopic Mass, Even Electron Ions

1 formula(e) evaluated with 1 results within limits (up to 50 best isotopic matches for each mass)

Elements Used:

C: 31-32 H: 51-52 N: 5-5 O: 6-6 S: 1-1 Si: 2-2

Minimum:				-1.5				
Maximum:	5.0	5.0		50.0				
Mass	Calc. Mass	mDa	PPM	DBE	i-FIT	Norm	Conf(%)	Formula
678.3187	678.3177	1.0	1.5	10.5	33.8	n/a	n/a	C31 H52 N5 O6 S Si2

O[6]-Methyl-2′-deoxyguanosine

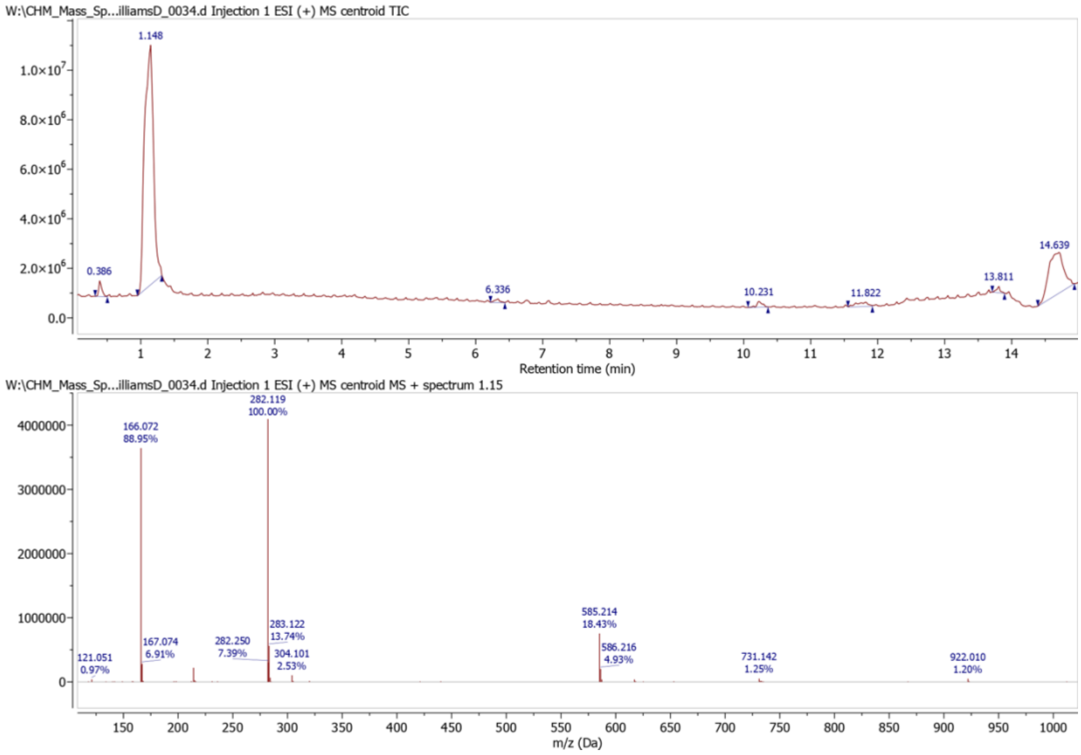

N[7]-Methyl-2′-deoxyguanosine

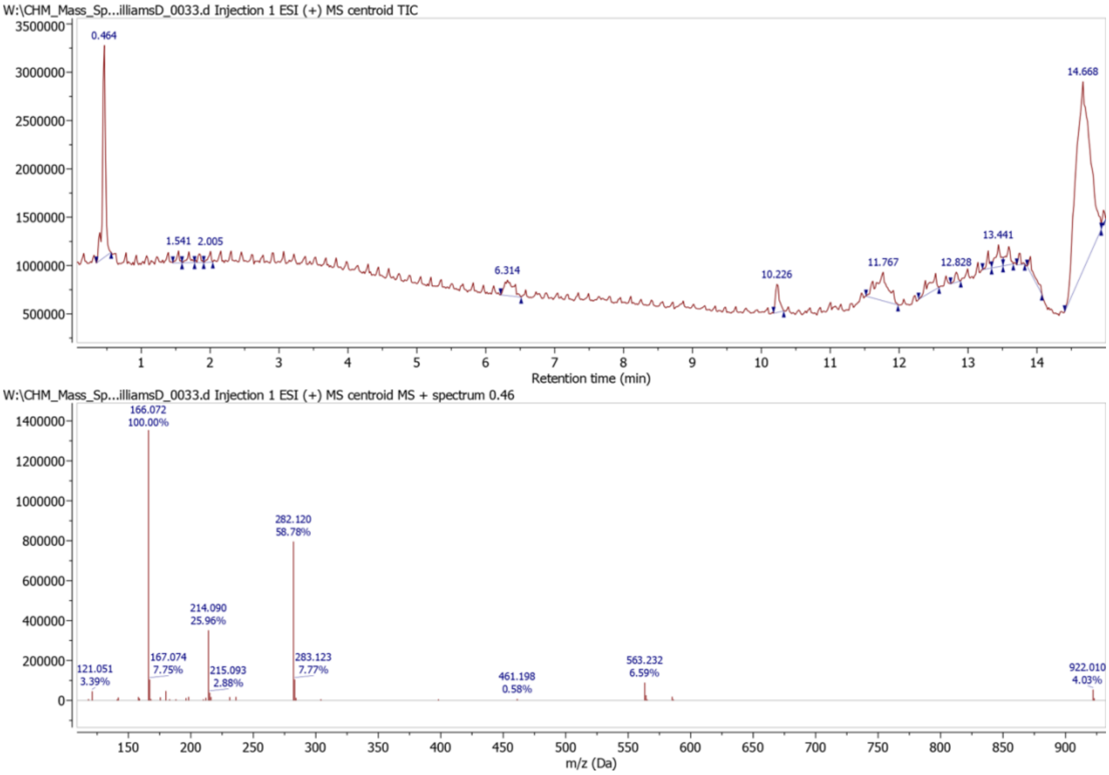

O^6-(Pterosin B)-2′-deoxyguanosine (O^6-PTBdG)

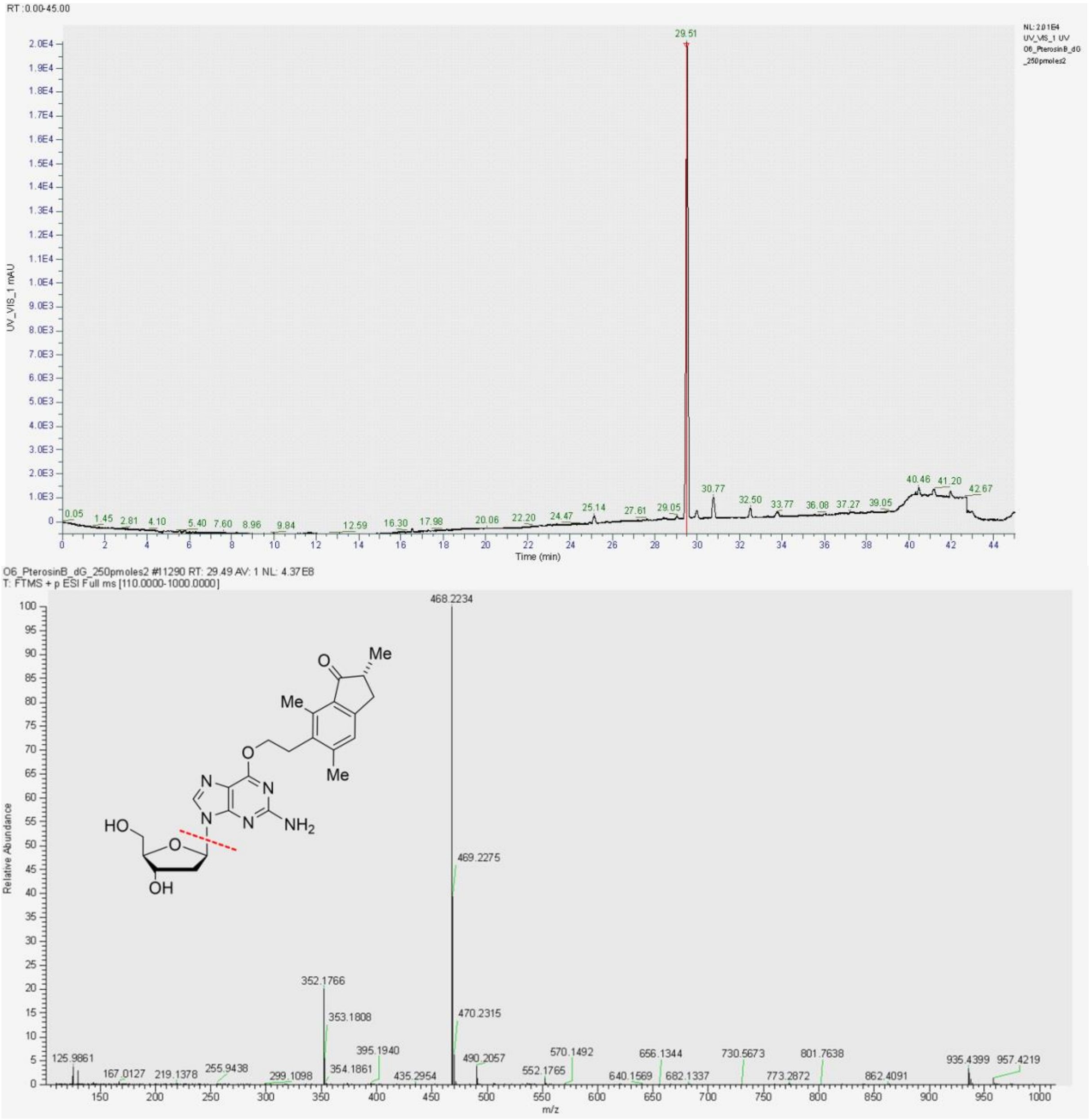

Formula	Calculated Mass	Target Mass	Double Bond Equivalence	Absolute Error (ppm)	Error (mDa)	Error (ppm)	Fitness
$C_{24}H_{29}N_5O_5$	468.22344	468.22541	13.0	4.23	1.97	4.23	1.000

N^7-(Pterosin B)-2′-deoxyguanosine N^7-PTBdG

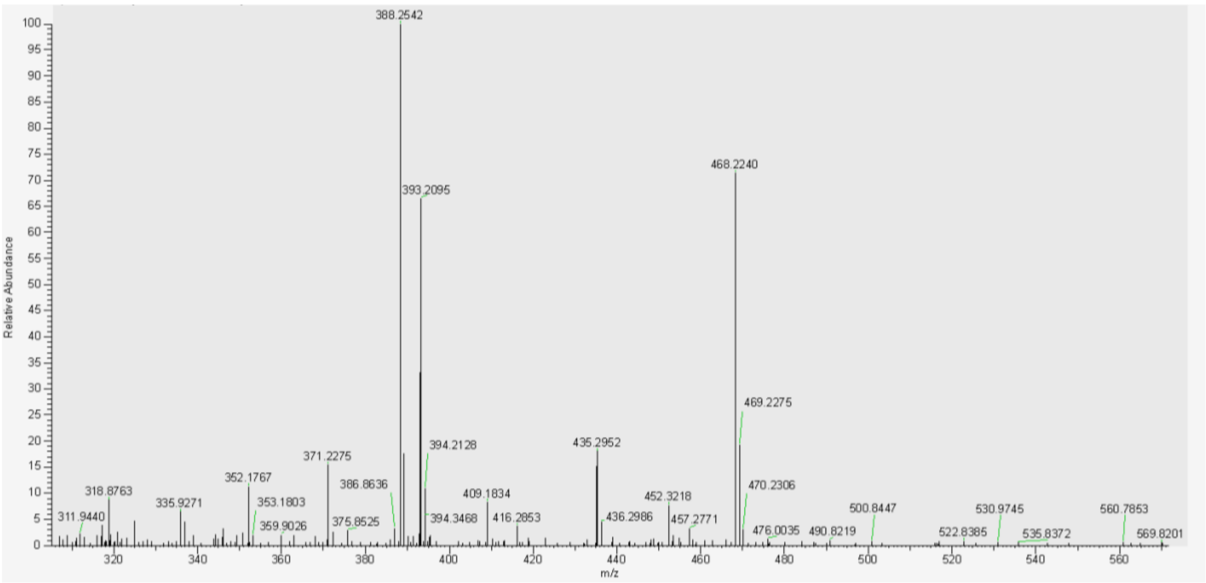

8, References

[1] M. Ojika, K. Wakamatsu, H. Niwa, K. Yamada, *Tetrahedron*, 1987, **43**, 5261-5274.
